# The moderation role of board independence change in the relationship between board characteristics, related party transactions, and financial performance

**DOI:** 10.1371/journal.pone.0279159

**Published:** 2022-12-15

**Authors:** Faozi A. Almaqtari, Najib H. S. Farhan, Hamood Mohammed Al-Hattami, Tamer Elsheikh

**Affiliations:** 1 Faculty of Business, Economics and Social Development, Universiti Malaysia Terengganu, Kuala Nerus, Terengganu, Malaysia; 2 Faculty of Business Strides, Arab Open University, A’ali, Saudi Arabia; 3 Faculty of Commerce and Economic, Department of Accounting, Hodeidah University, Al Hudaydah, Yemen; 4 Faculty of Commerce, Kafrelsheikh University, Kafrelsheikh, Egypt; University of Liverpool, UNITED KINGDOM

## Abstract

The present study examines the moderation effect of board independence change on the relationship between board characteristics, related party transactions and financial performance of Indian listed banks over 10 years from 2010 to 2019. While board size, independence, diligence, and remuneration were taken to represent board characteristics, all key personnel and subsidiaries’ transactions were considered measures for related party transactions. On the other hand, the financial performance of banks was measured by two accounting-based measures (return on assets and profit after tax) and two market-based measures (earning per share and Tobin Q). The results revealed that board independence change has a significant negative effect on financial performance. Further, the results indicated that board independence change moderates positively and significantly the relationship between related party transactions and financial performance. The findings also showed that board independence change had a moderating effect that significantly and negatively weakens board size and effectiveness, negatively influencing banks’ profitability. Unlike other studies, this study uniquely uses board independence change as a moderator between board characteristics, related party transactions, and several measures of banks’ financial performance. The limited research highlighting this issue, where Indian banks have encountered several challenges in the last few years, has motivated the present study to bridge the existing gaps in the strand literature. Therefore, this research opens useful insights and has beneficial implications for policymakers, bankers, financial analysts, and academicians.

## 1. Introduction

Prior research classified related party transactions (RPTs) into two contradicting streams based on agency [1–3] and efficient transaction viewpoints [4]. From the agency perspective, RPTs are a possible instrument for transferring a firm’s wealth to its related parties or vis versa [5]. Contradictory, the efficient transaction viewpoint considers RPTs as a tool that maximizes a firm’s value and minimizes transaction costs, especially in emerging markets [6]. Similarly, Bansal and Thenmozhi [5] indicated that RPTs could impact a firm’s destructive and enhanced value. Accordingly, a firm should balance both: avoiding destructive RPTs and promoting beneficial transactions. Given the destructive viewpoint, RPTs are prone to direct agency costs whereby managers or directors can profit at shareholders’ expense. Such opportunistic self-dealing behavior signals that investors’ needs are not a primary concern of the management [7,8].

Lins [9] indicates that insiders are tempted to divert resources to their interests in opportunistic behavior. According to agency theory, the board’s primary role is to oversee managerial behavior to control any power misappropriation of shareholders’ wealth [10] because abusive RPTs signify a clear signal of a self-dealing behavior which indicates more management-centric behavior, such as earnings management are more likely [8]. Accordingly, the expropriation of minority shareholders through self-dealing RPTs by dominant owners may be exacerbated by a lack of enforcement and a poor legal environment. The movement of resources between companies and associated parties boosts their gain [5]. In this context, the amendments incorporated in the Companies Act 2013 and listing requirements for public companies have brought better transparency in RPTs. According to the amendments, publicly listed firms are required mandatorily to disclose the transaction of RPTs with its related parties in their annual report, and minority shareholders are empowered to raise their concerns about RPTs of a firm by voting and/or by a nominated board member. Further, Islam [11] states that the listed companies are obliged to disclose the details of RPTs in their annual reports.

The current study aims to investigate the moderation effect of board independence change on the relationship between board characteristics, related party transactions, and financial performance of Indian listed banks. Firstly, we examine how RPTs affect banks’ financial performance (FP) and how the association between BC and RPTs influences FP. Secondly, we assess the moderation effect of board independence change on the relationship between BC, RPTs, and FP. We estimate these issues against financial performance that is measured by two accounting-based measures (Return on Assets (ROA) and Profit After Tax (PAT)) and two market-based measures (Earnings Per Share (EPS) and Tobin Q). In this comprehensive research model, we collectively examined the effect of RPTs and BC against FP of a sample comprising 38 Indian public and private banks from 2010 to 2019 using several statistical analysis tools. First, we estimated the impact of board characteristics and related party transactions on banks’ financial performance. Second, we estimated the moderation role of board independence change on the relationship between related party transactions and board characteristics on banks’ financial performance. Third, several robustness checks have been conducted using multiple tools of analysis. Finally, various steps and analysis tools have been adopted to test the sensitivity of the results in different scenarios.

Therefore, the present study contributes to the existing literature in different ways. Firstly, from a theoretical perspective, our study bridges the gap in research studies on the banking sector in India. The review on RPTs, BC, and BP studies in India is limited. Most empirical research on the topic of BC and its association with profitability has been confined primarily to manufacturing firms [12–18]. Evidence on this topic for the banking sector is absent in the Indian context. There are few studies examining the association between RPTs and the financial performance of banks in some developed countries. However, these countries’ unique institutional, legal, and financial settings differ from emerging countries, especially India. Accordingly, this potentially has practical and theoretical implications for other emerging economies. Secondly, the current study has a unique contribution as it investigates how all key personnel and subsidiaries as proxies for RPTs associate with board size, board composition, and board remuneration to influence banks’ financial performance in India. Thirdly, the current study investigates banks’ financial performance using two accounting-based measures (return on assets and profit after tax) and two market-based measures (earning per share and Tobin Q). The majority of prior studies adopt either accounting or market-based measures. Finally, the study assesses the moderating effect of board independence change on the relationship between related party transactions and board characteristics on banks’ financial performance, which has not been examined by prior research.

The current research is important as it investigates the link between board characteristics, related party transactions, and financial performance in the context of Indian banks. The banking system in India is evolving and emerging [19]. The development of the system is accompanied by a growing number of reported fraud cases from 2009 to 2022. The number of fraud cases has increased from 4,372 in 2009 to 9,103 in 2022 [20]. Further, the Indian economy is distinguished by the dominance of family business groups[21]. Despite these features, the Indian economy has weak functioning institutions [22], ineffective legal framework implementation [23], and a low penalty for corporate fraud [24]. This includes the country’s underdeveloped financial system, lax regulatory framework implementation and enforcement, concentrated ownership structure, and the phenomenon of corporate group connections [25]. According to Allen et al.[23], even though India has a legal framework based on common law that provides a solid regulatory framework for corporations, legislative implementation has been inadequate in practice. To this end, it is necessary to investigate this issue in the Indian context, which shapes the uniqueness of our study, considering financial institutions in India. Accordingly, the present study opens valuable insights into several issues related to the current practices in Indian banks in terms of RPTs and BC. Valuable insights are also offered to bankers, regulators, and policymakers for better performance of Indian banks and controlling the negative aspects of RPTs.

The rest of this research is organized as follows: section 2 provides a theoretical framework, section 3 reviews the existing literature, section 4 illustrates the research methodology, section 5 discusses the findings, and section 6 concludes the study.

## 2. Theoretical framework

### 2.1 Related party transactions under the Companies Act. 2013 in India

Corporate governance is one of the most often debated topics among academic and industry researchers in the present financial environment [26–32]. India is one of the countries that constantly monitors global trends in corporate governance and revises its regulatory framework regularly. In the Indian context, related party transactions are regulated under the Companies Act 2013, which has replaced the Companies Act 1956. Under the Companies Act 2013, the criteria for related party transactions can be split into four components: the identification of related parties, related party transactions, the approval process, and disclosure requirements [33]. The first component of the regulatory framework defines related party as directors and their relatives, a company in which a director, manager, or their relative is a partner, a body corporate whose board, managing director, or manager is accustomed to acting in accordance with the advice, to a public business in which a director or management is a director and owns more than 2% of the firm’s paid-up share capital with his relatives, a private company in which a director or manager is a member or director, directions or instructions a director or manager is accustomed to act, except if advice/ directions/ instructions are given in the professional capacity, directions or instructions of a director or manager, except if advice/ directions/ instructions are given in the professional capacity, any person on whose advice, any company which is a holding, subsidiary, or an associate company of such company, or a subsidiary of a holding company to which it is also a subsidiary, such other persons as may be prescribed.

The second component of the regulatory framework, according to the Companies Act of 2013, is the identification of relevant related party transactions. The Act in section 118 considers transactions related to the purchase, Sale of goods, selling, purchasing or disposing of any properties, supplying goods, leasing properties, the appointment of any agent for the acquisition or sale of commodities, materials, services, or property, the appointment of a related party to any office or profit-generating position inside the organization, underwriting the subscription of any securities or derivatives of the firm by its subsidiary or affiliate company. The third component of the regulatory framework is the approval of related party transactions. The Act in Section 188 (1) specifies the following requirements for related party transaction approval:

“*No firm shall engage into any contract or agreement with a related party with respect to purchase, Sale of goods*, *selling, purchasing or disposing any kind of properties, supplying goods, leasing properties, appointment of any agent for the acquisition or sale of commodities, materials, services, or property, appointment of a related party to any office or profit-generating position inside the organization, underwriting the subscription of any securities or derivatives of the firm by its subsidiary or affiliate company*, *unless the board of directors consents by resolution at a meeting of the Board and subject to such restrictions as may be stipulated. Finally, the fourth component of the regulatory framework, according to the Companies Act. of 2013 is disclosure. The Act stipulates in its section 188 (2), any arrangement / contract comes under section 188 (1) shall be disclosed in the board report along with justification*”.

Earlier studies from India reported that performance was negatively associated with the extent of RPTs but positively for stand-alone companies [34]. Prior research in India has not investigated RPTs in the context of Indian banks. However, very few studies have been conducted in India in this regard. For example, Wasan and Mulchandani [10] examined 182 firms listed on S&P BSE 500 from 2010 to 2018. Islam [11] utilized data from 322 firms for a period spanning from 2008 to 2015. Accordingly, there is a lack of research that assess RPTs in the context of financial institutions in India.

### 2.2 India’s banking sector reforms

Over the last two decades, extensive banking reform initiatives have been implemented to strengthen market institutions and give Indian banks more autonomy [35]. India had taken several liberalized policy initiatives, including financial sector reforms, in the early 1990s. The reforms aimed to restructure financial facilities, particularly the banking sector, to improve efficiency [36]. It responded by enacting a series of reforms that included reducing state-provided financing, privatizing banks, and general economic liberalization [37]. Gulati [35] indicated that banking reforms address a number of issues. First, the statutory pre-emption has been gradually reduced to make more resources available for commercial purposes. Second, the structure of administered interest rates has been gradually deconstructed. Third, the burden of directed sector lending has gradually been reduced by broadening the definition of priority sector lending and liberalizing lending rates on advances greater than INR 0.2 million. Fourth, entry regulations for domestic and foreign banks have been relaxed to increase competition in the banking sector. Fifth, policymakers implemented improved prudential standards for capital adequacy, asset classification, and income recognition that are consistent with international standards and increased disclosure levels. Sixth, in order to strengthen public sector banks, the Government of India recapitalized them to avert a financial crisis and increase their capital base to meet minimum capital adequacy norms. The various policy initiatives implemented during financial liberalization resulted in a number of changes in the banking industry, including improved asset quality, a decrease in non-performing assets (NPAs), the operation of private players, and the entry of foreign entities [36].

The major stream of commercial banking has dominated the Indian financial system. Its contribution to providing healthy financial facilities is significant. The changes in different regulatory environments and the diversified nature of ownership patterns are noteworthy in emphasizing the critical role of banking in the process of economic growth. Several changes in the banking system in terms of operational autonomy and ownership, such as collaborations, mergers and acquisitions, new banking services, and advances in information technology available to banks, are likely to improve banking performance and, thus, profitability [36]. Following the implementation of the reforms, many private sector banks were permitted to enter, and foreign banks were granted more liberal branch licensing policies. These liberalization measures have altered the competitiveness and efficiency of India’s commercial banks.

Along with increased competitiveness, the banking sector in India has seen a process of bank consolidation, followed by mergers and acquisitions, resulting in a reduction in the number of competitors and an increase in bank concentration [38]. The major stream of commercial banking has dominated the Indian financial system. Its contribution to providing healthy financial facilities is significant. Several changes in the Indian banking system in terms of operational autonomy and ownership, such as collaborations, mergers and acquisitions, new banking services, and advances in information technology available to banks, are likely to improve aggregate banking performance and, thus, profitability [36]. Public sector banks control about 70% of all bank assets [37].

## 3. Literature review

### 3.1 Banks’ financial performance

Several recent studies have investigated the profitability and financial performance of Indian banks [39–50]. These studies have used different proxies for measuring the financial and profitability of Indian banks. While Mukherjee [51], Mishra and Pradhan [42], Akhtar et al. [39], Al-Homaidi et al. [40], Almaqtari et al. [52], Chaki et al. [45], and Priyadarshini and Gomathi [53] used ROE, Saraswat [54] used return on investments and Akhtar et al. [39], Al-Homaidi et al. [40], and Sinha et al. [55] measured profitability using net interest margin. Kaur and Vij [41] used Tobin Q and economic value added to measure financial performance.

However, most of these studies have not assessed the relationship between related party transactions and banks’ profitability. For example, Priyadarshini and Gomathi [53] established a relationship between CSR and banks’ profitability. Further, some studies assessed the association between ownership and banks’ profitability [56], some other studies investigated banks specific factors and banks’ profitability [39,40,55,57]. on the other hand, Umasankar and Ashok [58] examined the impact of human resources on banks’ profitability, and Gaur and Mohapatra [48] assessed the effect of non-performing assets on banks’ profitability. None of these studies established a relationship between board attributes and related party transactions on Indian banks’ profitability. Kaur and Vij [41] linked corporate governance with the financial performance of Indian banks; however, the study utilized the corporate governance index. Further, different from prior research on banks’ profitability in India, this study examines four proxies of the financial performance of Indian banks: Return on net worth, return on capital employed, Earnings per share, and Profit after tax.

In addition, several studies have been conducted to examine different aspects of banks’ performance in many countries; Malaysia: [59]; Pakistan: [60]; Asian countries: [61]; Europe: [62]; Ghana: [63]; Russia: [64]; the US: [65]; Nigeria: [66]. None of these studies has investigated the relationship between related party transactions, board characteristics, and banks’ performance. While these studies have been conducted in developed and developing countries, it is argued that in emerging markets with different cultural, regulatory, corporate governance, and institutional contexts, voluntary compliance with corporate governance codes will differ from what has been observed in developed countries [67].

### 3.2 Related party transactions

Several studies indicate that financial scandals have occurred due to involvement in related party transactions [68,69]. Fooladi and Farhadi [70] advocate that prior studies suggest that most expropriation of firms’ resources occurs through related party transactions (RPTs). Agency costs could be increased opportunistically if related parties use their authorities to expropriate firms’ resources [70], and RPTs can be used to exploit corporate wealth [8,71]. Ming and Wong [72] provided evidence from 137 Chinese firms that firm value was significantly and negatively influenced by loans granted to related parties. Dahya et al. [73] reported that firms with RPTs have a greater value than firms with recourse to such transactions. Gordon et al. [74] revealed that RPTs from executive and non-executive directors were negatively and significantly linked with abnormal stock market yields in some US firms.

In the same context, Kohlbeck and Mayhew [8] suggested that negative yields were associated with transactions from firms’ directors, managers, and main shareholders. Several studies reported that insiders might exploit investors’ funds opportunistically for their benefit in the form of RPTs [1,3,9,71]. In the Indian context, according to Clause 49 of the listing agreement and Companies Act of 2013, businesses must have the materiality of interactions with related parties’ policies. All major RPTs must be approved by the audit committee and shareholders in advance by a special resolution [10]. Accordingly, board independent members act for the benefit of shareholders; they can mitigate any negative effect. Independent directors add value to a company by increasing accountability and providing objective judgment. A board with a higher proportion of outside directors has better management oversight. The board’s independence was found to influence several board decisions, including the negotiation of tender offers and the firing of non-performing CEOs [75]. Hence, to enhance board effectiveness and monitoring, we propose that there should be a higher proportion of independent board directors. If there is any change in the board, board independence change should be positive to increase the number of independent board members. Therefore, the following hypothesis has been formulated:

***H1*: *Board independence change moderates significantly and positively the association between related party transactions and banks’ financial performance***

### 3.3 Board of directors’ characteristics

In the literature, several studies discussed the effect of BC on banks’ performance with several parameters [13,15,65,76–82]. For example, Liang et al. [80] studied BC and bank performance in China, O’Connell et al. [81] examined the ’relationship between firm performance and board characteristics in Ireland.’ Berger et al. [15] assessed ’executive board composition and bank risk-taking in Germany’. Moreover, Al-Jaifi [61] explored the relationship between board gender diversity and environmental, social, and corporate governance performance in ASEAN banks’, and Titova [65] discussed whether ’board characteristics relevant for banking efficiency in the US.’ These studies’ findings confirm that BC plays a major role in banks’ performance. Further, multiple corporate governance variables have been assessed in several studies to determine how they influence the performance of banks [59,83]. Liang et al. [80] pointed out the positive impact of BC on bank performance. Moreover, Claessens and Yurtoglu [83] scientifically observed that governance practices are very beneficial to companies as they enable better access to various finance and debt instruments, reduce capital costs, improve operational effectiveness, and create a harmonious relationship with shareholders. Based on the above discussion, the study expects governance variables to have a positive and significant impact on Indian banks profitability.

Bhatia and Gulati [84] investigated the evolution of board practices in Indian banks through an index that included 14 BC from 2004 to 2017. The study indicated that the average score of the board index increased by 33% over time, marking a significant improvement in the board governance practices of banks. This improvement was mainly due to a significant shift in the sub-dimensions of board structures and board independence. Shukla et al. [85] investigated the effect of board size on the accounting returns and asset quality of 29 Indian banks listed on the National Stock Exchange from 2009 to 2016. The findings indicated that board size has a positive effect on ROA. However, it has an insignificant role in determining asset quality. Gupta and Mahakud [86] assessed the impact of chief executive officers’ personal characteristics on the performance of Indian commercial banks. The results concluded that CEO duality and professional qualification in the finance stream have a positive and significant effect on banks’ performance.

Concerning board size in India, Dey and Sharma [87] investigated the association between corporate governance and the financial performance of 10 public banks over seven years ending in 2019. The study revealed a significant and negative relationship between board size and banks’ performance as measured by ROA and ROE. Similarly, Shukla et al. [85] investigated the effect of board size on the accounting returns and asset quality of 29 Indian banks listed on the National Stock Exchange from 2009 to 2016. The findings indicated that board size has a positive effect on ROA; however, it has an insignificant role in determining asset quality. Similarly, Saravanan et al. [88] examined the influence of BC on 40 Indian banks’ performance. The findings revealed that the increase in board size has a significant relationship with better bank performance within both low and high board size ranges, but it is negatively linked with bank performance in the intermediate board size range.

Bhatia and Gulati [84] investigated the evolution of board practices in Indian banks through an index that included 14 BC from 2004 to 2017. The study indicated that the average score of the board index increased by 33% over time, indicating a significant improvement in the board governance practices of Indian banks. This improvement was mainly due to a significant shift in the sub-dimensions of board structures and board independence. Dey and Sharma [87] reported a significant negative relationship between board independence and Indian banks’ performance as measured by ROA and ROE. However, the study reported a positive association between the number of woman directors, executive directors, non-executive directors, and Indian banks’ performance measures. Abdul Gafoor et al. [76] indicated a significant association between board independence and a larger number of financial experts on the board and bank performance. However, the results revealed insignificant improvement in bank performance when the role of CEO and chairman were separated. Mayur and Saravanan [88] showed no relationship between board composition and performance. Gupta and Mahakud [86] concluded that CEO duality and the professional qualification of CEOs in finance have a positive and significant effect on Indian banks’ performance.

#### 3.3.1 Board size

Numerous studies have been conducted to study the relationship between board size and corporate performance [13,78,89–93]. While a research stream reports a significant positive relationship between board size and banks’ performance, another stream indicates a negative relationship. For example, using a sample of banking firms, Adams and Mehran [94] reported that board size is positively and significantly related to banks’ performance. Mayur and Saravanan [88] revealed that an increase in board size has a significant relationship with better bank performance within both low and high board size ranges. However, it is negatively linked with bank performance in the intermediate board size range. Abdul Gafoor et al. [76] indicated a significant association between board size and bank performance. Malik et al. [95] used a sample of 14 listed commercial banks in Pakistan from 2008–2012. They reported a significant positive relationship between board size and bank performance.

Contradictory, KyereboahColeman and Biekpe [96] examined the impact of board size among some other board characteristics on performance measures, namely ROA, Tobin Q, and Growth in sales of non-financial listed firms on the Ghana Stock Exchange during the period from 1990 to 2001. The results revealed that smaller board sizes are considered an effective drive for performance. Dey and Sharma [87] revealed a significant negative relationship between board size and banks’ performance. However, Ghosh and Ansari [78] showed that board size has an insignificant effect on performance, but it is indicated that board size matters in high-income areas. The results also reported that larger boards lead time-consuming meetings and are less conducive to performance.

Further, using a sample of the 50 largest Chinese banks from 2003 to 2010, Liang et al. [80] found that board size has a significantly negative impact on bank performance. To this end, as a result of inconsistent results of the relationship between board size and banks’ performance, several studies suggest that board independence should be considered in this regard [59,76,78,80,85,87,88,97,98]. This is to mitigate the negative effect of the decision process in larger board sizes and the absence of diversity and expertise in small board sizes. Hence, we propose that the increasing degree of board independence can play a significant and positive role between board size and banks’ performance. This is important, especially when there is a change in the board size. Accordingly, the following hypothesis has been proposed:

***H2*: *Board independence change moderates significantly and positively the association between board size and banks’ financial performance***

#### 3.3.2 Board diligence

Many studies have investigated the association between board meetings and banks performance in different countries; Saudi Arabia [99], China [80]; GCC [100]; Nigeria [101–103] Malaysia [59,104], and India [27,28,41,56,87,88,105–108]. The majority of these studies indicated a positive association between board meetings and banks’ performance. Dey and Sharma [87] investigated the association between corporate governance and the financial performance of 10 public banks over seven years ending in 2019. The study revealed a significant and negative relationship between board meetings, board committees and board independence, and banks’ performance as measured by ROA and ROE. Abdul Gafoor et al. [76] examined the effect of board structure characteristics on the performance of 36 scheduled Indian commercial banks from 2001 to 2014. The results indicate a significant association between board independence, the number of board meetings, and a larger number of financial experts on the board and bank performance. Mayur and Saravanan [88] examined the influence of BC on 40 Indian banks’ performance. The findings revealed no relationship between board meetings and board composition and performance. Hence, we hypothesize that a positive board independence change enhances the positive relationship between board meetings and banks’ performance. Therefore, the following hypothesis has been developed:

***H3*: *Board independence change moderates significantly and positively the association between meetings (diligence) size and banks’ financial performance***

#### 3.3.3 Board remuneration

In India, very few researches have been conducted to assess directors’ and managerial remuneration. For example, Aggarwal and Ghosh [109] aimed to investigate the effect of directors’ remuneration on the intrinsic and extrinsic value of Indian firms. From an investor’s viewpoint, the findings revealed an insignificant association between a higher directors’ remuneration and a better firm’s performance. However, from the accounting viewpoint, the results reported a positive link between the two indicating that directors’ remuneration contributes to firms’ intrinsic value but does not add significantly to firms’ extrinsic value. Bhattarai and Negi [110] indicated that managerial remunerations, among several other variables, are statistically significant factors in firms’ profitability for the years 2004, 2008, 2012, and 2014. Kang and Nanda [111] examined the effect of firms’ performance, size, ownership structure, BC, and other company characteristics on the disclosure of managerial remuneration of 134 Indian-listed firms from 2003 to 2012. The results indicated that the disclosure of managerial remuneration is significantly associated with the existence of a remuneration committee and company size.

Further, there was a substantial development and an increase in the disclosure of remuneration over the years. Das and Mohanty [112] assessed managers’ remuneration to ensure dividend stability while maintaining investment and financing decisions. The results showed that the average managerial remuneration, among other variables such as past dividends, firm size, return on net worth, firm maturity, and debt to equity, significantly impacts the regular dividend-paying behavior of Indian firms. Existing literature on directors’ remuneration is predominantly carried out in developed countries. However, there are limited studies in this regard in India. Lee and Isa [59] found clear evidence of a positive relationship between director remuneration and performance in 21 banks from 2003 to 2011. Furthermore, the results revealed that director remuneration significantly affects performance. Directors’ remuneration was found to be related to the percentage of independent directors but not to duality or the percentage of director share ownership. The results also indicated that foreign banks outperform domestic banks despite their directors’ relatively lower pay. Accordingly, we assume that a positive board independence change can mitigate the negative association between directors’ remuneration and banks’ performance. Hence, we hypothesize that:

***H4*: *Board independence change moderates significantly and positively the association between directors’ remuneration and banks’ financial performance***

## 4 Methodology

### 4.1. Data collection and sampling

The current research relied on secondary data from the Prowess Q database. The data covers ten years from 2010 to 2019. The sample size is made up of 38 banks that are publicly traded on the Bombay Stock Exchange (BSE). The current research is limited to commercial banks in India. The sample includes the majority of public-sector banks. These banks were chosen based on their BSE listing status and data availability for the period covered by this study. Furthermore, the current analysis solely looks at commercial banks, leaving out regional-rural banks and urban-rural cooperative banks. Almaqtari et al. [52] reported that there are 56 regional rural banks and 43 listed banks, in addition to 27 public and 46 international banks. They stated that public-sector banks account for a significant percentage of the banking sector assets. More specifically, the assets of both the "National and State Bank of India and its Associates" account for around 70% of the banking system’s total assets. As a result, the current research includes 38 banks listed on the BSE, consisting of private, international, and public banks.

### 4.2. Operational definition of variables

**Fig 1:**
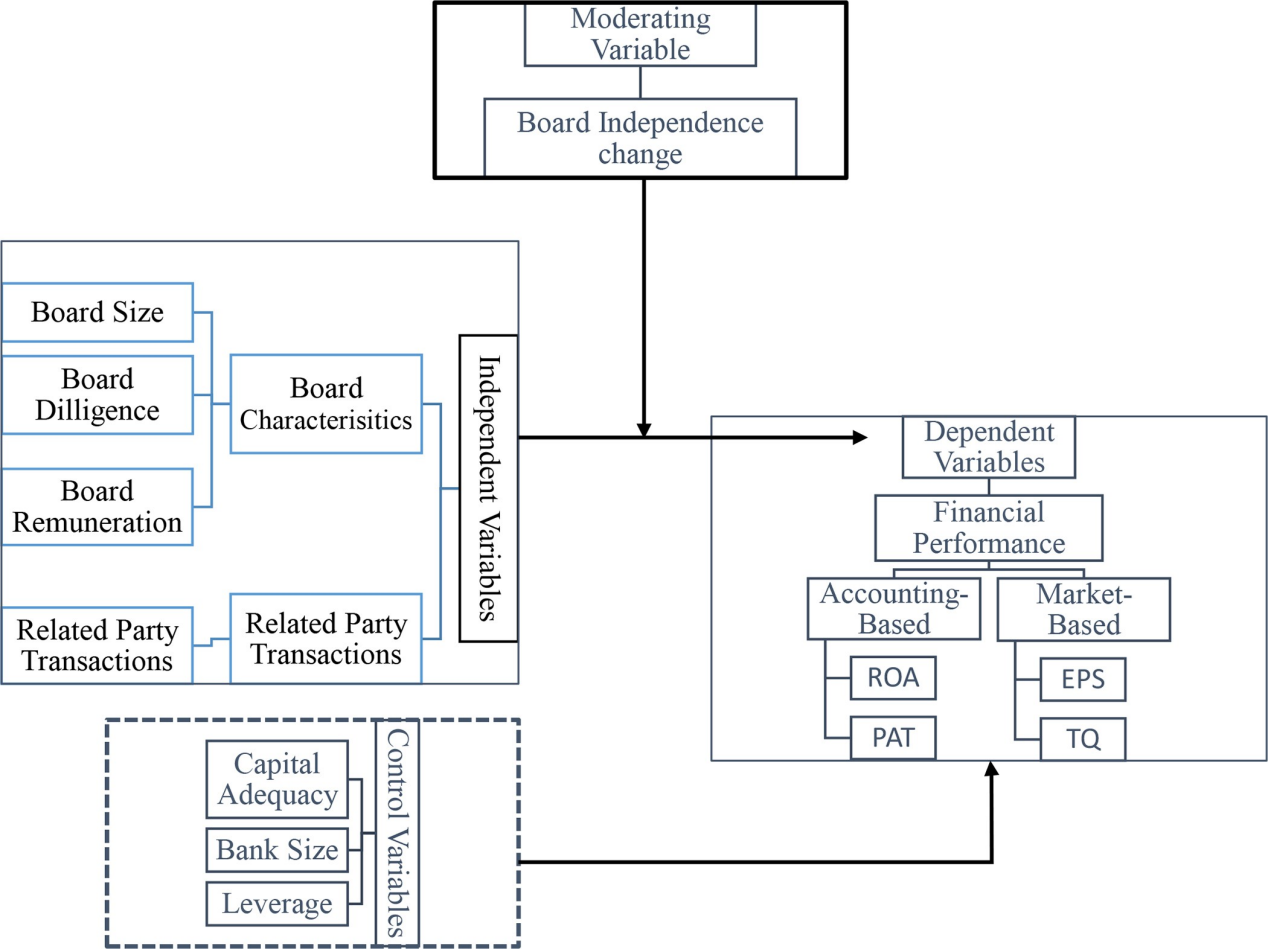
Research framework.

Fig 1 provides the research framework that includes BC and related party transactions as independent variables, bank specifics as control variables, financial performance as the dependent variable, and board independence change as a moderating variable. Among board characteristics, the board size, independence, diligence, and remuneration have been selected to be our predictors of financial performance. Although some studies have investigated board characteristics using board or corporate governance index [41,113–117], a specific conclusion to what extent each characteristic contributes to banks’ performance cannot be drawn. We followed a research call by a systematic review conducted by Almaqtari et al. [28], who indicated that these characteristics are frequently investigated by studies in the context of Indian firms. Further, several studies considered board size [16,76,78,80,85,87,88,97,98,118] board independence [76,87,97,119], board meetings [87,99,104,107,108,120], and board remuneration [16,59,107]. These studies affirm that such board characteristics significantly influence banks’ performance.

In the Indian context, Jain and Agarwalla [121] state that the Indian economy is characterized by a thriving capital market and a sizable presence of domestic and foreign institutional investors. Furthermore, the Indian economy is notable for the dominance of family business groups [21]. Some institutional factors influence the relationship between auditors and managers and the incentives for earnings management in India.

These include the country’s underdeveloped financial system, the regulatory framework’s comparatively weak implementation and enforcement, the country’s traditionally concentrated ownership structure, and the phenomenon of corporate group connections [25]. Accordingly, board independence is considered an effective monitoring factor that enhances board effectiveness. Gong et al. [122] indicated that controlling shareholders’ tunneling behavior may be caused by a lack of independent directors on the board. Independent directors are thought to be more objective and effective at monitoring management [122]. The lack of effective markets and formal institutions in emerging countries adds to concerns about the effectiveness of independent directors [122]. Thus, independence change is considered by the current study because it is an efficient driver that improves board effectiveness. The financial performance of banks as the dependent variable was measured by two accounting-based measures (return on assets and profit after tax) and two market-based measures (earning per share and Tobin Q). Many researchers believe that market-based measures appear more appropriate than accounting-based measures in measuring and presenting the performance of business units. However, several uncontrollable factors influence the market-based measure [123]. Hutchinson and Gul [124] advocate that account-based measures are preferable to market-based ones to evaluate the performance of business units to reflect the results of management actions. As a result, the present study adopts a comprehensive approach to include both accounting-based and market-based measures. Table 1 shows the operational definition of the study’s variables.

**Table 1:** Operational definition of the variables of the study.

Variable		Symbol	Formula
**Dependent Variables**			
Accounting-Based Measures	Return on Assets	ROA	$\frac{\mathrm{Net}\;\mathrm{Income}}{Total\;Assets}$
	Profit after tax	PAT	Net Profit After Tax
Market-Based Measures	Earnings per share	EPS	$\frac{Net\;Income}{Number\;of\;outstanding\;shares}$
	Tobin Q	TQ	$\frac{Total\;Market\;value}{Total\;Asset\;Value}$
**Independent Variables**			
Related party transactions		RPT	Amounts of transactions with all key personal parties + amounts of transactions with all subsidiaries
Board of directors’ size		BS	It is the total number of board of directors of a bank
Board independence		BI	$\frac{Number\;of\;independent\;directors}{Total\;number\;of\;board\;of\;directors}$
Board diligence		BD	$\frac{Total\;number\;of\;meetings\;attended\;by\;a\;board\;member}{Total\;number\;of\;board\;meetings\;held\;during\;amembr\prime s\;tenure\;in\;a\;year}$
Remuneration		REM	Cash remuneration, which included base salary and annual bonus
**Control Variables**			
Leverage		LEV	Is the ratio of total debts to equity
Capital Adequacy		CA	$\frac{Tier\;1\;Capital+Tier\;2\;Capital}{Risk\;Weighted\;Assets}$
Bank Size		SIZE	Is the natural logarithm of total assets
**Moderating Variable**			
Board independence change		BICH	Is the ratio of board independence change from a year t and years t-1
**Other Control Variables for Sensitivity Analysis**			
Indian Accounting standards		Ind.ASs	Is a dummy variable of 1 for the years 2017 onwards in which Ind. ASs are implemented and 0 otherwise
Return on capital employed		ROCE	Net profit after tax/ Total issued capital

### 4.1.Econometric tools and model specification

While some studies utilized functional linear forms [125], some other researchers used panel data analysis [126–128]. Further, other studies utilized both GMM and linear regression models [60,129–133]. However, the present study adopts several stages and tools of analysis to estimate the effect of RPTs and BC on FP. In the first analysis stage, we estimated the impact of board characteristics and related party transactions on banks’ financial performance. In the second step, we evaluated the moderation role of board independence change on the relationship between related party transactions and board characteristics on banks’ financial performance.

Consequently, in the third stage of analysis, several robustness checks were conducted using multiple analysis tools, including Two-Stage Least Square (2SLS) regression, Generalized Method of Moments (GMM), lag of independent variables, and Heckman selection test. In the last stage of our analysis, we provided some additional analysis to test the sensitivity of the results. In this stage, we estimated the impact of related party transactions and board characteristics on banks financial performance of private and public banks. Then, we used an alternative measure of financial performance to test the sensitivity of the results provided in the preceding steps. Finally, we estimated the impact of related party transactions and board characteristics on financial performance by considering the effect of India Accounting Standards (Ind. ASs), in which we conducted the pre-post analysis.

To investigate the effect of BC, RPTs and bank-specifics on FP of Indian listed banks, the following models are designed.


$${FP}_{it}=\alpha +\beta_{1}\sum_{j=1}^{4}C_{it}+\beta_{2}\sum_{j=1}^{2}X_{it}+\beta_{3}\sum_{j=1}^{3}Y_{it}+\varepsilon_{it}$$ (1)

Where *C*_*it*_ represents the board of directors’ characteristics, *X*_*it*_ indicates related party transaction, Y_it_ refers to bank specifics and i, t and *ε*_*it*_ measure the individual effect, the temporal effect, and the stochastic error, respectively. Where;

$$\sum_{j=1}^{4}C_{it}=\alpha +\beta_{1}{BS}_{it}+\beta_{2}{BI}_{it}+\beta_{3}{BD}_{it}+\beta_{4}{REM}_{it}+\varepsilon_{it}$$ (2)

$$\sum_{j=1}^{2}X_{it}=\alpha +\beta_{1}{KRPT}_{it}+\beta_{2}{SRPT}_{it}+\varepsilon_{it}$$ (3)

$$\sum_{j=1}^{3}Y_{it}=\alpha +\beta_{1}{LEV}_{it}+\beta_{2}{CA}_{it}++\beta_{3}{SIZE}_{it}+\varepsilon_{it}$$ (4)

Accordingly, *FP* is functioned by $\sum_{j=1}^{4}\;C_{it}$ as an indicator of BC, $\sum_{j=1}^{2}\;X_{it}$ as measures of related party transactions, and $\sum_{k=1}^{3}\;Y_{t}+\varepsilon_{it}$ as some measures of bank-specific factors. Based on these equations, the following main model is formulated:

$${FP}_{it}({ROA}_{it}/{PAT}_{it}/{EPS}_{it}/{TQ}_{it})=\alpha +\beta_{1}{BS}_{it}+\beta_{2}{BI}_{it}+\beta_{3}{BD}_{it}+\beta_{4}{REM}_{it}+\beta_{5}{RPT}_{it}+\beta_{6}{LEV}_{it}+\beta_{7}{CA}_{it}+\beta_{8}{SIZE}_{it}+\varepsilon_{it}$$ (Model 1)

## 5. Analysis and discussion

### 5.1.Descriptive statistics

The results in Table 2 show that BS has an average of 14 members, a minimum of 5 members, a maximum of 22 members, and a standard deviation of 3.26, indicating a variation among the board size of the selected banks. BI reveals that board independence has a minimum of 20% of their board members as board independent members, with a maximum of 92% and an average of 36%. BD also shows that the average diligence of board meetings is 70%, with a minimum of 31% and a maximum of 100%. Further, REM has an average of 26.83 (Million) with a standard deviation of 51.15, indicating a variation in board remuneration among the sampled banks. In the same context, the results of Big-4 indicate that only 8% of the sampled banks are audited by Big-4 companies. RPT by both key personnel and subsidiaries have a maximum of 140 and 2.21 (Millions), respectively, with a minimum of nil and standard deviation of 7.21 and 0.14, indicating a significant variation among the banks. Moreover, the results show that bank-specific factors, LEV, CA, and SIZE, have an average of 1.31, 0.01, and 6.11, respectively.

**Table 2:** Descriptive statistics.

Variables	Minimum	Maximum	Mean	Median	Std. Dev.
ROA	-4.76	10.22	0.53	0.65	1.42
PAT	-151.90	210.70	9.30	5.35	35.39
EPS	-106.50	243.90	18.32	11.10	40.09
TQ	0.45	12.68	0.80	0.69	0.77
RPT	0.00	2.21	0.03	0.00	0.14
BS	5.00	22.00	13.45	13.00	3.26
BI	0.20	0.92	0.36	0.38	0.28
BD	0.31	1	0.70	0.69	0.14
REM	0.00	410.00	26.83	7.68	51.15
LEV	0.01	5.82	1.31	1.17	0.89
CA	0.00	0.37	0.01	0.00	0.02
SIZE	3.39	7.57	6.11	6.20	0.59
BICH	0.00	0.75	0.44	0.23	0.12
Ind.ASs	0.00	1.00	0.30	0.00	0.46
ROCE	-42.43	20.53	2.55	4.90	9.79

Note: ROA is Return on Assets, PAT is Profit after tax, EPS is Earnings per share, TQ is Tobin Q, RPT is Related party transactions, BS is Board of directors’ size, BI is Board independence, BD is Board diligence, REM is Remuneration, LEV is Leverage, CA is Capital Adequacy, SIZE is Bank Size, BICH is Board independence change, Ind.ASs is Indian Accounting standards, and ROCE is Return on capital employed.

### 5.2.Correlation analysis

Table 3 provides a correlation analysis among the study variables. The results show that all financial performance measures negatively correlate with RPT, BICH, and Ind.ASs. While ROA has a negative correlation with SIZE and BS, PAT has a negative relationship with LEV, CA, and SIZE. Similarly, EPS exhibits a negative correlation with LEV and CA. All other independent and control variables exhibit positive and negative relationships; however, the maximum correlation is observed in the case of the relationship of BD and BS (-0.56, þ < 0.01), indicating a negative correlation and that board diligence is low with a larger board size. Importantly, the results reveal that BI has a significant negative relationship (þ < 0.01) with related party transactions and SIZE. This signifies that RPT is high in banks that have low board independence. Further, it indicates that board independence is low in large banks.

**Table 3:** Correlation matrix.

Var.	BS	BI	BD	REM	RPT	LEV	CA	SIZE	BICH	Ind.ASs	ROA	PAT	EPS	TQ
BS	1.00													
BI	-0.31***	1.00												
BD	-0.56***	0.28***	1.00											
REM	-0.04	0.29***	0.16***	1.00										
RPT	0.04***	-0.07***	-0.05	0.02***	1.00									
LEV	-0.06	-0.06	-0.18***	0.13***	0.05***	1.00								
CA	-0.24***	-0.05	-0.04	-0.04	0.01	0.24***	1.00							
SIZE	0.47***	-0.37***	-0.13***	0.17***	0.12***	0.04	-0.46***	1.00						
BICH	-0.12**	-0.06	-0.07	-0.03	0.08***	0.04	0.41***	-0.18***	1.00					
Ind.ASs	-0.07	0.02	0.03	0.23***	0.08	-0.08	-0.03	0.20***	0.01	1.00				
ROA	-0.03	0.12***	0.13***	0.17***	-0.05***	0.07	0.08	-0.24***	-0.02***	-0.49***	1.00			
PAT	0.03	0.18***	0.16***	0.11***	-0.07***	-0.08	-0.05	-0.22***	-0.03***	-0.48***	0.94***	1.00		
EPS	0.14***	0.02	0.04	0.10***	-0.03***	-0.02	-0.07	0.12***	-0.03***	-0.34***	0.53***	0.53***	1.00	
TQ	0.13***	0.07	0.12***	0.50***	-0.01***	0.04	-0.11***	0.26***	-0.06***	-0.24***	0.58***	0.51***	0.56***	1.00

Note

*** p<0.01

** p<0.05

* p<0.1. All variables are defined in Table 1.

### 5.3.The impact of BC, RPTs, and bank-specific on FP of Indian banks

To estimate this relationship, fixed and random effect models have been tested, leading to choosing random effect models over fixed ones based on the Hausman test. Jaisinghani (2016) stated that certain assumptions should be fulfilled to estimate the random effect model, which are as follows:

$$u_{it}∼N(0,\sigma_{u}^{2})$$

$$\alpha_{i}∼N(0,\sigma_{\alpha }^{2})$$

$$\lambda_{t}∼N(0,\sigma_{\lambda }^{2})$$

$$Cov(u_{it},u_{js})=\sigma_{u}^{2},\;if=i=j$$

$$Cov(u_{it},u_{js})=0,\;Otherwise$$

$$Cov(\alpha_{i},\alpha_{j})=\sigma_{\alpha }^{2},\;if=i=j$$

$$Cov(\alpha_{i},\alpha_{j})=0,\;Otherwise$$

$$Cov(\lambda_{t},\lambda_{s})=\sigma_{\alpha }^{2},\;if=t=s$$

$$Cov(\lambda_{t},\lambda_{s})=0,\;Otherwise$$

$$Cov(X_{it},u_{js})=Cov(X_{it},\alpha_{i})=Cov(X_{it},\lambda_{s})=Cov(u_{it},\alpha_{j})=Cov(u_{it},\lambda_{t})=Cov(\alpha_{i},\lambda_{t})=0\forall i.j,s,t$$ (5)

The results in Table 4 demonstrate an estimation of OLS regression models for the impact of BC, related party transactions, and bank specifics on the financial performance of Indian banks. The results across the financial performance measures are fit, indicated by the probability value of 1% (þ < 0.01). The adjusted R squared for all models varied between 15% to 30%, indicating that the variables of the models explain about 15% to 30% of the variability of those models.

**Table 4:** The impact of BC, RPTs, and bank specifics on FP of Indian banks.

Variables	Model (1a)	Model (1b)	Model (1c)	Model (1d)
	ROA	PAT	EPS	TQ
C	2.523***	4.309***	5.273***	-7.430***
	0.663	1.578	2.648	11.360
BS	2.204***	1.273***	2.150***	7.504**
	0.361	0.264	0.725	0.907
BI	-2.860***	-3.243***	-1.724***	-2.880**
	6.205	8.128	5.332	5.160
BD	3.800***	8.463***	3.158*	3.060**
	1.134	3.046	1.531	1.770
REM	0.114***	0.119**	0.120**	0.258***
	0.003	0.004	0.004	0.011
RPT	0.149***	0.166***	0.128***	0.102***
	0.022	0.026	0.021	0.013
LEV	4.081**	1.011	-2.913	40.642**
	1.970	1.031	2.759	8.200
CA	-86.292	-76.080*	-241.866*	-28.921
	8.209	6.626	22.876	2.830
SIZE	-2.489***	-1.378***	-3.643***	5.760**
	8.066	7.899	9.126	1.050
R-squared	0.173	0.194	0.388	0.181
Adjusted R-squared	0.146	0.167	0.297	0.154
F-statistic	6.371	7.327	4.254	6.748
Prob(F-statistic)	0.000	0.000	0.000	0.000
Observations	380	380	380	380

Note

*** p<0.01

** p<0.05

* p<0.1. All variables are defined in Table 1.

${ROA}_{it=}\alpha +\beta_{1}{BS}_{it}+\beta_{2}{BI}_{it}+\beta_{3}{BD}_{it}+\beta_{4}{REM}_{it}+\beta_{5}{RPT}_{it}+\beta_{6}{LEV}_{it}+\beta_{7}{CA}_{it}+\beta_{8}{SIZE}_{it}+\varepsilon_{it}\;\mathrm{Model}\;(1a)$.

${PAT}_{it=}\alpha +\beta_{1}{BS}_{it}+\beta_{2}{BI}_{it}+\beta_{3}{BD}_{it}+\beta_{4}{REM}_{it}+\beta_{5}{RPT}_{it}+\beta_{6}{LEV}_{it}+\beta_{7}{CA}_{it}+\beta_{8}{SIZE}_{it}+\varepsilon_{it}\;\mathrm{Model}\;(1b)$.

${EPS}_{it=}\alpha +\beta_{1}{BS}_{it}+\beta_{2}{BI}_{it}+\beta_{3}{BD}_{it}+\beta_{4}{REM}_{it}+\beta_{5}{RPT}_{it}+\beta_{6}{LEV}_{it}+\beta_{7}{CA}_{it}+\beta_{8}{SIZE}_{it}+\varepsilon_{it}\;\mathrm{Model}\;(1c)$.

${TQ}_{it=}\alpha +\beta_{1}{BS}_{it}+\beta_{2}{BI}_{it}+\beta_{3}{BD}_{it}+\beta_{4}{REM}_{it}+\beta_{5}{RPT}_{it}+\beta_{6}{LEV}_{it}+\beta_{7}{CA}_{it}+\beta_{8}{SIZE}_{it}+\varepsilon_{it}\;\mathrm{Model}\;(1d)$.

The results reveal that all BC exhibit a significant impact on the four measures of financial performance. While BS, BD, and REM have a significant positive impact on the four measures of financial performance, BI exhibits a significant but negative impact on financial performance. At the same time, BS exhibits a significant impact on ROA, PAT, and EPS at the level of 1% (þ < 0.01), and it has a significant influence on TQ at the level of 5% (þ < 0.05). This indicates that the financial performance of Indian banks positively associates with board size, and the larger the bank’s board size, the greater the bank’s profitability. This is consistent with the argument that a larger board size may include better diversity, positively contributing to better financial performance. Dey and Sharma [87] reveal a significant and negative relationship between board size and banks’ performance as measured by ROA and ROE. Similarly, Abdul Gafoor et al. [76] indicate a significant association between board size and bank performance. Shukla et al. [85] also suggest that board size has a positive effect on ROA. However, it has an insignificant role in determining asset quality. Similarly, Saravanan et al. [88] found that the increase in board size has a significant relationship with better bank performance within both low and high board size ranges, but it is negatively linked with bank performance in the intermediate board size range. Contradictory, Ghosh and Ansari [78] provide that board size has an insignificant effect on performance; however, it is indicated that board size matters in high-income areas and larger boards lead to time-consuming meetings and are less conducive to performance.

The results also reveal that BI has a significant but negative influence on ROA, PAT, and EPS at the level of 1% (þ < 0.01), and it has a significant effect on TQ at the level of 5% (þ < 0.05).

This may indicate a limited contribution of BI to the profitability of the Indian banks. The negative sign does not reveal a negative relationship. However, it signifies either a low percentage of BI on the board or the effective role of board independent members. This is consistent with Bhatia and Gulati [84], who indicated that the average score of the board index increased by 33% over time, indicating a significant improvement in the board governance practices of Indian banks. This improvement in board practices of Indian banks was mainly due to a significant shift in the sub-dimensions of board structures and board independence. Dey and Sharma [87] also report a significant and negative relationship between board independence and Indian banks’ performance as measured by ROA and ROE. Further, Abdul Gafoor et al. [76] indicate a significant association between board independence and bank performance. However, Mayur and Saravanan [88] revealed no relationship between board composition and performance.

The results also reveal that BD has a positive influence on ROA and PAT at the level of 1% (þ < 0.01), EPS at the level of 10% (þ < 0.10), and TQ at the level of 5% (þ < 0.05). This signifies a positive relationship between profitability and board diligence. This is consistent with Dey and Sharma [87], who revealed a significant negative relationship between board meetings and banks’ performance as measured by ROA and ROE. Contradictory, Ghosh and Ansari [78] show that larger boards lead to time-consuming meetings and are less conducive to performance. However, Mayur and Saravanan [88] reveal that there is no relationship between board meetings and board composition and performance.

In the same vein, REM shows a positive effect on ROA and TQ at the level of 1% (þ < 0.01); however, it has a positive impact on PAT and EPS at the level of 5% (þ < 0.05). This reveals a positive association between board remuneration and the profitability of Indian banks. This is similar to the argument of [109]. They revealed that—from an investor’s viewpoint- there is an insignificant association between a higher directors’ remuneration and a better firm’s performance. However, from the accounting viewpoint, the results report a positive link between the two, indicating that directors’ remuneration contributes to firms’ intrinsic value but does not add significantly to firms’ extrinsic value. Similarly, Bhattarai and Negi [110] indicated that managerial remunerations, among several other variables, are statistically significant factors in firms’ profitability. Das and Mohanty [112] show that the average managerial remuneration has a significant impact on regular dividend-paying behavior of Indian firms.

Concerning related party transactions, they demonstrate a significant positive impact on the financial performance of Indian banks. While it has a significant positive impact on ROA and PAT at the level of 1% (þ < 0.01), it has a significant positive impact at the level of 10% (þ < 0.10) on EPS and at the level of 5% (þ < 0.05) on TQ. This is consistent with the argument that RPTs can be used as a way to exploit corporate wealth (Cheung et al., 2006; Kohlbeck & Mayhew, 2017). Consistently, Ming and Wong [72] provided evidence from 137 Chinese firms that firm value was significantly and negatively influenced by loans granted to related parties. Gordon et al. [74] revealed that RPTs from executive and non-executive directors were negatively and significantly linked with abnormal stock market yields in some US firms. In the same context, Kohlbeck and Mayhew [8] suggested that negative yields were associated with transactions from firms’ directors, managers, and main shareholders. Several studies reported that insiders might exploit investors’ funds opportunistically for their own benefit in the form of RPTs [1,3,71]. Dahya et al. [73] reported that firms that have RPTs have a greater value than other firms that have recourse to such transactions.

Accumulating evidence on RPTs is heterogeneous [2]. Prior research indicates that RPTs are not always a mechanism for fraud and that not all types of RPTs are associated with fraud cases [134]. Although research has shown that firms use RPTs to manage earnings to conceal their performance [2], RPTs have also been shown to impact firm performance negatively. According to Chen et al. [135], firms’ post-IPO long-term underperformance is an unintended consequence of using RPTs to boost performance in the pre-IPO period. Ryngaert and Thomas [136] consistently indicate that RPTs may be advantageous to firm performance. Skinner and Sloan [137] suggest that firms with poor performance have a greater incentive to manage earnings through RPTs. Some studies have found that not all types of RPTs are associated with earnings management [134,136]. Others have found them to be innocuous rather than indicative of opportunistic behaviour [136]. El-Helaly [2] argues that the association between RPT and earnings management, when measured using an accruals-based measure, might be insignificant or even negative. Contradictory, Ahraony et al. [138] discover evidence that RP sales and non-payment of RP loans are used opportunistically to manage earnings. This supports our findings that the positive association between RPTs and a bank’s performance could signify earnings management. Accordingly, we investigated the effect of board characteristics in this relationship to evaluate the efficiency of the corporate governance mechanism applied by the banks. According to El-Helaly [2], corporate governance mechanisms can mitigate RPTs’ negative impact on firm value and performance. RPTs are associated with controlling shareholders’ power, such as insider ownership, voting rights, and the ability to influence independent board members through increased compensation. Regarding banks’ specifics, LEV exhibits a significant positive impact on ROA and TQ at the level of 5% (þ < 0.05). CA shows a significant negative impact on ROA and EPS at 10% (þ < 0.10). However, SIZE exhibits a significant negative impact on ROA, PAT, and EPS at the level of 1% (þ < 0.01) and a significant positive impact on TQ at the level of 5% (þ < 0.05).

### 5.4. The moderating effect of board independence change on the relationship between RPTs and BC on FP of Indian banks

In this study, BICH is considered a moderator and is measured by the difference between BI in years t and t-1. Further, BICH is scaled by BS to evaluate BICH in terms of its relationship with total BS. The following formula is used to measure BICH:

$$\frac{BICH}{BS_{it}}=\frac{{BI}_{it}-{BI}_{it-1}}{\sum_{j=1}{BS}_{ii}}$$ (6)

where *BICH*_*it*_ = *BI*_*it*_−*BI*_*it*−1_.

In order to assess the moderating effect of BC ($\sum_{j=1}^{4}\;C_{it}+\varepsilon_{it}$), RPT $\left(\sum_{k=1}^{3}\;X_{it}+\varepsilon_{it}\right)$ and bank-specific factors ($\sum_{k=1}^{3}\;Y_{it}+\varepsilon_{it}$) on *FP*, the following regression model are estimated:

$${FP}_{it}({ROA}_{it}/{PAT}_{it}/{EPS}_{it}/{TQ}_{it})=\alpha +\beta_{1}{BS}_{it}+\beta_{2}{\left({BS}_{}\;x\;{BICH}_{}\right)}_{it}+\beta_{3}{BICH}_{it}+\beta_{4}{BD}_{it}+\beta_{5}{\left({BD}_{}\;x\;{BICH}_{}\right)}_{it}+\beta_{6}{BREM}_{it}+\beta_{7}{\left({REM}_{}\;x\;{BICH}_{}\right)}_{it}+\beta_{8}{RPT}_{it}+\beta_{9}{\left({RPT}_{}\;x\;{BICH}_{}\right)}_{it}+\beta_{10}{LEV}_{it}+\beta_{11}{\left({LEV}_{}\;x\;{BICH}_{}\right)}_{it}+\beta_{12}{CA}_{it}+\beta_{13}{\left({CA}_{}\;x\;{BICH}_{}\right)}_{it}+\beta_{14}{SIZE}_{it}+\beta_{15}{\left({SIZE}_{}\;x\;{BICH}_{}\right)}_{it}+\varepsilon_{it}$$ (Model 2)

The results in Table 5 provide an analysis of the moderating effect of BICH between BC, RPT, and bank specifics on the one hand and FP on the other. As far as RPT is concerned, the results show that BICH moderates positively and significantly the relationship between RPT and FP across all models. This moderation effect is significantly positive at the level of 1% (þ < 0.01) in all models. This indicates that BICH strengthens the impact of RPT on FP, which could be a sign for earning management. This leads to accepting H1, which indicates that BICH moderates positively and significantly the relationship between RPTs and banks’ performance. This is consistent with prior studies that investigated the relationship between related party transactions and banks’ performance [8,70–74].

**Table 5:** Moderating role of board independence change.

Variables	Model (2a)	Model (2b)	Model (2c)	Model (2d)
	ROA	PAT	EPS	TQ
*C*	8.203***	6.434**	5.225*	6.400***
	0.846	0.014	04.674	0.700
*BSB*	3.738***	2.182***	4.919**	7.832***
	1.150	0.598	1.899	0.058
*BSx BICH*	-4.388**	-2.518***	-6.272**	-9.398***
	1.584	0.811	3.181	2.538
*BICH*	-37.335***	-17.810***	-4.578**	-2.310***
	12.067	4.964	30.415	1.689
*BD*	55.457*	27.815**	73.768	5.600***
	32.240	13.953	61.659	2.930
*BDx BICH*	-55.050	-27.815	-93.045	-28.200***
	45.350	20.784	96.004	24.370
*REM*	12.002***	2.020**	24.837***	9.459
	3.461	0.089	11.581	3.285
*REMx BICH*	1.020***	1.178***	3.387***	0.911**
	0.028	0.590	0.001	0.028
*RPT*	2.204***	1.273**	1.611***	1.917***
	0.355	0.259	0.289	2.561
*RPTx BICH*	3.959***	1.917***	9.971***	0.057***
	6.030	2.561	5.088	0.365
*LEV*	14.732**	9.717***	9.933	73.862
	5.217	2.934	7.069	42.521
*LEVx BICH*	-8.836***	-14.021***	-20.816**	-38.654
	6.406	3.555	10.357	22.321
*CA*	-196.299*	-86.021	-76.854	-42.700**
	112.811	53.793	34.010	65.200
*CAx BICH*	-3.639	-8.484**	-9.758	-42.700**
	8.520	17.095	16.446	78.600
*SIZE*	-51.762***	-21.074***	-65.903**	-95.400***
	10.664	5.221	23.959	77.450
*SIZEx BICH*	-60.078***	-22.911***	-15.707***	-23.100***
	13.894	6.244	3.936	12.720
R-squared	0.199	0.227	0.177	0.203
Adjusted R-squared	0.162	0.193	0.154	0.197
F-statistic	16.158	18.178	8.057	12.533
Prob(F-statistic)	0.000	0.000	0.000	0.000
Observations	380	380	380	380

Note

*** p<0.01

** p<0.05

* p<0.1. All variables are defined in Table 1.

${ROA}_{it=}\alpha +\beta_{1}{BS}_{it}+\beta_{2}{\left({BS}_{}\;x\;{BICH}_{}\right)}_{it}+\beta_{3}{BICH}_{it}+\beta_{4}{BD}_{it}+\beta_{5}{\left({BD}_{}\;x\;{BICH}_{}\right)}_{it}+\beta_{6}{BREM}_{it}+\beta_{7}{\left({REM}_{}\;x\;{BICH}_{}\right)}_{it}+\beta_{8}{RPT}_{it}+\beta_{9}{\left({RPT}_{}\;x\;{BICH}_{}\right)}_{it}+\beta_{10}{LEV}_{it}+\beta_{11}{\left({LEV}_{}\;x\;{BICH}_{}\right)}_{it}+\beta_{12}{CA}_{it}+\beta_{13}{\left({CA}_{}\;x\;{BICH}_{}\right)}_{it}+\beta_{14}{SIZE}_{it}+\beta_{15}{\left({SIZE}_{}\;x\;{BICH}_{}\right)}_{it}+\varepsilon_{it}\;\mathrm{Model}\;(2a)$

${PAT}_{it=}\alpha +\beta_{1}{BS}_{it}+\beta_{2}{\left({BS}_{}\;x\;{BICH}_{}\right)}_{it}+\beta_{3}{BICH}_{it}+\beta_{4}{BD}_{it}+\beta_{5}{\left({BD}_{}\;x\;{BICH}_{}\right)}_{it}+\beta_{6}{BREM}_{it}+\beta_{7}{\left({REM}_{}\;x\;{BICH}_{}\right)}_{it}+\beta_{8}{RPT}_{it}+\beta_{9}{\left({RPT}_{}\;x\;{BICH}_{}\right)}_{it}+\beta_{10}{LEV}_{it}+\beta_{11}{\left({LEV}_{}\;x\;{BICH}_{}\right)}_{it}+\beta_{12}{CA}_{it}+\beta_{13}{\left({CA}_{}\;x\;{BICH}_{}\right)}_{it}+\beta_{14}{SIZE}_{it}+\beta_{15}{\left({SIZE}_{}\;x\;{BICH}_{}\right)}_{it}+\varepsilon_{it}\;\mathrm{Model}\;(2b)$

${EPS}_{it=}\alpha +\beta_{1}{BS}_{it}+\beta_{2}{\left({BS}_{}\;x\;{BICH}_{}\right)}_{it}+\beta_{3}{BICH}_{it}+\beta_{4}{BD}_{it}+\beta_{5}{\left({BD}_{}\;x\;{BICH}_{}\right)}_{it}+\beta_{6}{BREM}_{it}+\beta_{7}{\left({REM}_{}\;x\;{BICH}_{}\right)}_{it}+\beta_{8}{RPT}_{it}+\beta_{9}{\left({RPT}_{}\;x\;{BICH}_{}\right)}_{it}+\beta_{10}{LEV}_{it}+\beta_{11}{\left({LEV}_{}\;x\;{BICH}_{}\right)}_{it}+\beta_{12}{CA}_{it}+\beta_{13}{\left({CA}_{}\;x\;{BICH}_{}\right)}_{it}+\beta_{14}{SIZE}_{it}+\beta_{15}{\left({SIZE}_{}\;x\;{BICH}_{}\right)}_{it}+\varepsilon_{it}\;\mathrm{Model}\;(2c)$

${TQ}_{it=}\alpha +\beta_{1}{BS}_{it}+\beta_{2}{\left({BS}_{}\;x\;{BICH}_{}\right)}_{it}+\beta_{3}{BICH}_{it}+\beta_{4}{BD}_{it}+\beta_{5}{\left({BD}_{}\;x\;{BICH}_{}\right)}_{it}+\beta_{6}{BREM}_{it}+\beta_{7}{\left({REM}_{}\;x\;{BICH}_{}\right)}_{it}+\beta_{8}{RPT}_{it}+\beta_{9}{\left({RPT}_{}\;x\;{BICH}_{}\right)}_{it}+\beta_{10}{LEV}_{it}+\beta_{11}{\left({LEV}_{}\;x\;{BICH}_{}\right)}_{it}+\beta_{12}{CA}_{it}+\beta_{13}{\left({CA}_{}\;x\;{BICH}_{}\right)}_{it}+\beta_{14}{SIZE}_{it}+\beta_{15}{\left({SIZE}_{}\;x\;{BICH}_{}\right)}_{it}+\varepsilon_{it}\;\mathrm{Model}\;(2d)$.

The results show that BS, as moderated by BICH, has a significant negative effect across the models. It has a significant negative effect at the level of 1% (þ < 0.01) for PAT and TQ. It also demonstrates a significant negative impact at the level of 5% (þ < 0.05) for ROA and EPS. This indicates that BICH negatively moderates the relationship between BS and FP, which means that the change of board independent members weakens BS and diversity, negatively influencing banks’ profitability. The results also reveal that board independence change has a significant negative effect on financial performance across all models at the level of 1% (þ < 0.01) for ROA, PAT, and TQ. It also has a significant negative effect at 5% (þ < 0.05) for EPS. This indicates that BICH weakens the FP of the sampled banks across all models. This leads to rejecting H2, which states that BICH moderates significantly and positively the relationship between board size and banks’ performance. This could be because smaller board size is highly correlated with banks’ performance in India and a negative board independence change, which led to this negative moderating effect. The results of the present study are consistent with those of [28,41,56,87,88,107,120].

The results also reveal that BICH does not significantly moderate the relationship between BD and FP except in the case of TQ. This leads to rejection H3, which implies that BICH does not moderate the relationship between boar meetings and banks’ performance. This is consistent with [41,59,100,104,108,107,139]. Similarly, the results show a significant positive moderating effect of BICH between REM and FP across all models.

This effect is significant at the level of 1% (þ < 0.01) in the case of ROA, PAT, and EPS. However, it is significant at the level of 5% (þ < 0.05) in the case of TQ. Accordingly, H4 is accepted, indicating that BICH moderates the relationship between board remuneration and banks’ performance. Bhattarai and Negi [110], Kang and Nanda [111], Das and Mohanty [112], and Lee and Isa [59] also indicate similar findings. Finally, the results show that the moderating effect of BICH does not significantly moderate the relationship between bank specifics and FP across all models. This demonstrates a significant negative effect in the case of LEV and SIZE, indicating that board independence change is greater in larger banks and banks with greater leverage; this change affects FP negatively.

### 5.5. Generalized method of moments estimation

Several researchers indicate that GMM is better for tackling endogeneity. Abdul Gafoor et al. [76] suggest that in any BC analysis, the main issue is the endogeneity of board characteristics. This estimates that OLS regression is inconsistent and biased. Accordingly, the present study estimates the results using GMM estimation to tackle possible endogeneity and heterogeneity problems. Gupta and Mahakud [86] indicate that conducting GMM analysis solves the heterogeneity and endogeneity issues. Heterogeneity problems can be tackled by the first differences in order to avoid the individual effect that makes the estimation unbiased. Saona [132] indicates that problems associated with individual heterogeneity justify the utilization of GMM.

On the other hand, the endogeneity issues can be solved through GMM, which contains the lagged regressors as instruments. Further, Arellano and Bond [140] argue that GMM offers an advantage of orthogonal conditions among the lags of the regressors or explanatory variables in the model to enable additional instruments. Thus, the following GMM models are designed:

$${FP}_{it}({ROA}_{it}/{PAT}_{it}/{EPS}_{it}/{TQ}_{it})=\beta_{0}+\beta_{1}{FP}_{it-1}+\sum_{j=1}^{4}\;\delta_{j}C_{it}+\sum_{j=1}^{2}\;\delta_{j}X_{it}+\sum_{k=1}^{3}\;\theta_{k}Y_{t}+\eta_{i}+\mu_{t}+\varepsilon_{it}$$ (7)

Where *X*_*it*_ indicates the vector of the related party transactions, *C*_*t*_ is the vector that signifies board characteristics, *Y*_*t*_ represents bank specifics, *η*_*i*_, *μ*_*t*_ and εit indicate the individual effect, the temporal effect, and the stochastic error, respectively. Accordingly,

$$\sum_{j=1}^{4}\;\delta_{j}C_{it}=\delta_{1}{BS}_{it}+\delta_{2}{BI}_{it}{+\delta }_{3}{BD}_{it}+\delta_{4}{REM}_{it}$$ (8)

$$\sum_{j=1}^{2}\;\delta_{j}X_{it}=\delta_{1}{KRPT}_{it}+\delta_{2}{SRPT}_{it}$$ (9)

$$\sum_{j=1}^{3}\;\theta_{k}Y_{t}=\theta_{1}{LEV}_{it}+{+\theta_{2}{CA}_{it}++\theta_{3}{SIZE}_{it}}_{}$$ (10)

Accordingly, the following model is estimated:

$${FP}_{it}({ROA}_{it}/{PAT}_{it}/{EPS}_{it}/{TQ}_{it})=\beta_{0}+\beta_{1}{FP}_{it-1}+\delta_{1}{BS}_{it}+\delta_{2}{BI}_{it}++\delta_{3}{BD}_{it}++\delta_{4}{REM}_{it}++\delta_{5}{RPT}_{it}+\theta_{1}{LEV}_{it}+{\theta_{2}{CA}_{it}++\theta_{3}{SIZE}_{it}}_{}{+\eta }_{i}+\mu_{t}+\varepsilon_{it}$$ (Model 3)

The results in Table 6 reveal that BC exhibit a similar effect on FP compared to the OLS regression results in Table 4. All BC except for BI reveal a significant positive effect on FP; however, BI shows a significant negative effect across the four models. Further, GMM results show that RPT has a significant positive effect on FP, indicating robust results findings.

**Table 6:** GMM Estimation.

Models	Model (3a)	Model (3b)	Model (3c)	Model (3d)
Variables	ROA	PAT	EPS	TQ
LagDV.	0.658***	0.675***	0.675***	0.846***
	0.088	0.072	0.098	0.139
C	26.510**	10.500***	-171.8**	-67.000*
	44.730	21.140	67.330	135.000
BS	3.954***	1.934***	3.698**	4,664**
	1.529	0.576	1.885	55.021
BI	-20.410***	-5.474**	-37.940**	-29.145***
	13.830	1.299	24.140	82.327
BD	74.98**	31.89**	48.370*	33.927***
	38.810	15.230	47.400	12.452
REM	1.289***	6.210***	5.287**	7. 221**
	0.021	2.821	1.227	2.927
RPT	2.486***	4.279**	6.327**	5. 1270***
	0.873	1.492	2.378	1.924
LEV	8.679***	3.279**	9.573*	9.223*
	2.943	1.361	5.611	4.214
CA	-28.900**	-190.200**	79.304**	-59. 132
	47.452	31.301	26.652	71. 427
SIZE	-22.88***	-10.18***	-6.487**	-25.046***
	37.957	23.433	11.040	46. 129
R1	0.807	0.832	0.611	0.514
R2	0.829	0.918	0.435	0.376
Sargant	0.000	0.000	0.000	0.000

Note

*** p<0.01

** p<0.05

* p<0.1. All variables are defined in Table 1.

${ROA}_{it}=\beta_{0}+\beta_{1}{ROA}_{it-1}+\delta_{1}{BS}_{it}+\delta_{2}{BI}_{it}++\delta_{3}{BD}_{it}++\delta_{4}{REM}_{it}++\delta_{5}{RPT}_{it}+\theta_{1}{LEV}_{it}+{\theta_{2}{CA}_{it}++\theta_{3}{SIZE}_{it}}_{}{+\eta }_{i}+\mu_{t}+\varepsilon_{it}\;\mathrm{Model}\;(3a)$

${PAT}_{it}=\beta_{0}+\beta_{1}{PAT}_{it-1}+\delta_{1}{BS}_{it}+\delta_{2}{BI}_{it}++\delta_{3}{BD}_{it}++\delta_{4}{REM}_{it}++\delta_{5}{RPT}_{it}+\theta_{1}{LEV}_{it}+{\theta_{2}{CA}_{it}++\theta_{3}{SIZE}_{it}}_{}{+\eta }_{i}+\mu_{t}+\varepsilon_{it}\;\mathrm{Model}\;(3b)$

${EPS}_{it}=\beta_{0}+\beta_{1}{EPS}_{it-1}+\delta_{1}{BS}_{it}+\delta_{2}{BI}_{it}++\delta_{3}{BD}_{it}++\delta_{4}{REM}_{it}++\delta_{5}{RPT}_{it}+\theta_{1}{LEV}_{it}+{\theta_{2}{CA}_{it}++\theta_{3}{SIZE}_{it}}_{}{+\eta }_{i}+\mu_{t}+\varepsilon_{it}\;\mathrm{Model}\;(3c)$

${TQ}_{it}=\beta_{0}+\beta_{1}{TQ}_{it-1}+\delta_{1}{BS}_{it}+\delta_{2}{BI}_{it}++\delta_{3}{BD}_{it}++\delta_{4}{REM}_{it}++\delta_{5}{RPT}_{it}+\theta_{1}{LEV}_{it}+{\theta_{2}{CA}_{it}++\theta_{3}{SIZE}_{it}}_{}{+\eta }_{i}+\mu_{t}+\varepsilon_{it}\;\mathrm{Model}\;(3d)$

However, there is a slight change in the effect of BC in which LEV, CA, and SIZE exhibit greater significance compared to the results of OLS in Table 4. This could be due to some multicollinearity issues associated with the estimation of OLS regression. Overall, the findings of GMM estimation provide consistent and robust findings. This is also indicated by the model fit results in which all R1 and R2 values are insignificant (þ > 0.05) [141,142].

### 5.6.Robustness check

In the previous two sections, we investigated the direct impact of BC, RPT, and bank specifics on FP. Further, we investigated the moderating effect of BICH on the effect of BC, RPT, and BS on FP. This estimation, however, may have some endogeneity problems. Accordingly, we apply a two-stage least square (2SLS) estimation to tackle and account for endogeneity issues that may arise in the earlier estimations. We also conduct a generalized method of moment (GMM) to deal with multicollinearity and endogeneity issues. Further, we apply the lag of explanatory variables and Heckman’s two-stage estimation for more rigorous estimation of endogeneity problems and robust findings.

#### 5.6.1. Two-stage least square regression

Guest [143] indicates that endogeneity problems may arise in situations where firm-specific indicators are influenced by board structure, and both board structure and firm-specific indicators are jointly investigated by unobservable heterogeneity.

Hence, to control the endogeneity issues in this study, we follow Guest [143] and Mulherin [144] by creating bank type dummy variable. Nadarajah et al. [145] indicate that corporate governance variables may have possible endogeneity issues with leverage. In our study, we create two groups of private and public banks to control for bank type effect. In order to address this, we follow Yermack [146] and re-estimate the direct effect models in an instrumental variables’ framework, using lagged values of the dependent variables as instruments for current values. Several studies used 2SLS regressions to tackle the endogeneity problems (e.g., AL-Qadasi et al. (2019); (Stewart & Cairney, 2019)). Guest [143] also indicates that another endogeneity problem is reverse causality, whereby firm-specific explanatory variables are determined by board structure rather than vice versa. In particular, Yermack [146] found that board size and the percentage of outsiders negatively impact financial performance. We follow the following equation to address the endogeneity issues:

$${FP}_{it=}\beta_{0}+\beta_{1}y_{it}+\beta_{2}Z_{it}+\varepsilon_{it}$$ (11)

While banks’ specific variables (*y*_*it*_ = *LEV*_*it*_, *CA*_*it*_, *and SIZE*_*it*_) are considered endogenous variables, BC variables (*Z*_*it*_ = *BS*_*it*_, *BI*_*it*_, *BD*_*it*_, *and REM*_*it*_) are treated as exogenous variables. We use the lagged variables of the dependent variables and the fitted values of the main models as instrumental variables where *y*_*it*_ = *π*_0_+*π*_1_*Z*_*it*_+*υ*.

The results in Table 7, columns 1 (Model 1a) to 4 (Model 1d) provide 2SLS estimation for the direct model (Model 1), and columns 5 (Model 2a) to 8 (Model 2d) present the results of 2SLS for the moderating effect (Model 2).

**Table 7:** Two-stage least square regressions.

Models	Model (1a)	Model (1b)	Model (1c)	Model (1d)	Model (2a)	Model (2b)	Model (2c)	Model (2d)
Variables	ROA	PAT	EPS	TQ	ROA	PAT	EPS	TQ
*C*	21.54	6.07	-12.71***	-31.65***	-17.60**	-7.09**	-4.41***	-6.79***
	9.64	2.618	4.20	12.11	87.79	39.21	25.9	33.167
*BS*	2.258***	1.333***	9.298***	11.713**	7.984***	3.707***	11.02***	2.797***
	0.482	0.236	2.709	3.526	2.12	0.947	3.04	0.524
*BSx BICH*					-10.674**	-4.023*	-15.452***	-5.486**
					9.463	4.227	13.570	4.146
*BICH*	-9.254**	-2.443***	-17.9***	-10.950**	-7.137**	-3.654***	-8.092**	-1.558**
	4.762	2.332	24.12	7.145	19.430	12.287	22.425	4.198
*BD*	45.67***	25.22***	220.9***	33.755**	178.6***	81.01***	36.9***	47.739***
	10.76	5.267	63.3	16.139	48.6	21.71	19.68	17.122
*BDx BICH*					63.151	28.212	17.241	1.6448*
					22.055	9.696	9.172	0.997
*REM*	6.158**	1.138**	-3.407**	12.708***	-2.267*	5.077**	3.477**	2. 250***
	2.760	0.358	1.567	6.646	1.177***	1.228***	1.687**	0.475*
*REMx BICH*					-0. 251	-0.062	-0. 287	1.347
					0. 33	0. 148	0. 474	1.461
*RPT*	2.480***	-6.721**	1.046**	-0. 454**	-0.001**	-4.345***	-0. 152***	-0.024**
	1.430	6.998	7.427	0.013	9.045	4.045	0.013	0.040
*RPTx BICH*					0.311***	0.133**	0.465**	74.94**
					0.277	0.124	0.397	121.1
*LEV*	11.70***	5.296***	113.5***	1.296	87.36***	37.96***	115.7***	15.043***
	3.266	1.599	25.72	4.901	19.43	8.677	27.86	4.799
*LEVx BICH*					1.531	0.444	2.104	53.5
					1.305	0.583	1.872	221.7
*CA*	-141.1**	-113.1***	-1,054***	11.785***	-1.298**	-739.3***	-1.427*	-26.337***
	66.46	32.54	389.7	4.069	15.921	26.2	85.5	63.389
*CAx BICH*					9.018**	13.485*	34.126**	9.2388***
					36.581	4.025	12.006	3.098
*SIZE*	-16.34***	-8.105***	-19.12**	-12.185***	-31.99***	-15.86***	-17.6**	7.096***
	2.712	1.328	14.37	19.347	9.888	4.416	14.18	4.191
*SIZEx BICH*					-30.999*	-2.787	-52.149	-3.497*
					67.658	30.220	97.019	2.997
R-squared	0.19	0.31	0.23	0.34	0.32	0.29	0.36	0.312

Note

*** p<0.01

** p<0.05

* p<0.1. All variables are defined in Table 1.

The results in Table 7 provide robust and consistent results with those presented in Table 5 and Table 4. We, therefore, conclude that earlier OLS estimations presented in Table 4 and Table 5 are robust across the estimations of 2SLS models since they demonstrate consistent and robust results to our OLS findings.

#### 5.6.2. Lag of independent variables

Table 8 presents an OLS estimation with lagged independent variables. We follow Ullah et al. [147] and Kang and Nanda [111], who apply lagged independent variables for their governance variables. Bennouri et al. [148] also indicate that applying lagged independent variables is a viable tool for tackling endogeneity problems. We apply one-year-lagged independent variables, and the following model is estimated:

$${FP}_{it}({ROA}_{it}/{PAT}_{it}/{EPS}_{it}/{TQ}_{it})=\alpha +\beta_{1}{BS}_{it-1}+\beta_{2}{BI}_{it-1}+\beta_{3}{BD}_{it-1}+\beta_{4}{REM}_{it-1}+\beta_{5}{RPT}_{it-1}+\beta_{6}{LEV}_{it-1}+\beta_{7}{CA}_{it-1}+\beta_{8}{SIZE}_{it-1}+\varepsilon_{it}$$ (Model 4)

**Table 8:** Lagged independent variables.

Models	Model (4a)	Model (4b)	Model (4c)	Model (4d)	Model (4a)	Model (4b)	Model (4c)	Model (4d)
Variables	ROA	PAT	EPS	TQ	ROA	PAT	EPS	TQ
*C*	121.121***	56.533***	-28.307***	-50.750**	59.094***	27.445***	-91.300**	-45.280***
	25.462	11.166	45.944	88.430	17.225	9.062	56.424	79.190
*BS*	0.954***	0.351***	0.283**	82.594***	0.741**	0.440***	2.796***	9.875*
	0.192	0.088	0.486	48.777	0.325	0.126	0.623	5.103
*BSx BICH*					-3.375**	-6.514***	-8.487***	-7.221***
					10.711	15.611	31.786	20.018
*BICH*	-1.412***	-1.577**	-2.787***	-26.080***	-13.800***	-50.715**	-17.516**	-12.218***
	2.844	1.612	9.687	44.687	46.600	71.270	28.400	34.679
*BD*	12.101***	3.440**	-23.724**	51.007**	4.586***	2.730**	3.004**	63.433**
	3.447	1.221	13.027	28.375	3.135	1.437	0.993	29.631
*BDx BICH*					103.200	50.030	18.800	29.550*
					92.200	28.820	43.510	16.520
*REM*	2.257***	3.254**	2.548**	1.879	3.279**	3.736***	2.228***	4.289***
	0.521	1.687	0.254	0.441	1.021	2.682	0.420	1.452
*REMx BICH*					3.257**	1.290***	2.469**	1.116**
					0.001	0.198	0.415	1.034
*RPT*	4.521***	1.276**	0.824**	1.171***	3.456***	3.249**	2.498**	4.054**
	1.221	0.369	0.207	1.012	1.224	0.927	0.879	1.027
*RPTx BICH*					0.039***	0.014**	0.300**	1.486**
					0.011	0.005	0.129	81.973
*LEV*	2.500**	0.460	-5.872**	81.939*	3.743***	0.255	2.488	2.181**
	0.892	0.478	3.004	46.269	1.010	0.453	2.995	0.412
*LEVx BICH*					-11.930**	-12.781***	-13.500***	-22.000*
					19.580	34.594	34.520	54.000
*CA*	-21.131***	-12.953***	-49.855	-36.800***	-10.407***	-14.134***	-13.335***	-45.600***
	33.718	22.137	262.622	73.110	31.446	38.623	36.379	76.600
*CAx BICH*					37.020***	11.000**	35.000	39.218***
					9.220	24.400	14.000	12.000
*SIZE*	-21.278***	-9.606***	-35.122***	24.396	-9.929***	-4.958***	9.079	-9.190
	4.567	1.964	8.885	14.987	3.242	1.579	10.593	28.797
*SIZEx BICH*					-32.082	-22.002	-9.110	19.000
					55.170	54.830	24.140	31.000
R-squared					0.263	0.265	0.221	0.28
Adjusted R-squared					0.252	0.245	0.19	0.27
F-statistic					4.340	4.398	3.455	6.099
Prob(F-statistic)					0.000	0.000	0.000	0.000

Note

*** p<0.01

** p<0.05

* p<0.1. All variables are defined in Table 1.

${ROA}_{it=}\alpha +\beta_{1}{BS}_{it-1}+\beta_{2}{BI}_{it-1}+\beta_{3}{BD}_{it-1}+\beta_{4}{REM}_{it-1}+\beta_{5}{RPT}_{it-1}+\beta_{6}{LEV}_{it-1}+\beta_{7}{CA}_{it-1}+\beta_{8}{SIZE}_{it-1}+\varepsilon_{it}\;(\mathrm{Model}\;4a)$.

${PAT}_{it=}\alpha +\beta_{1}{BS}_{it-1}+\beta_{2}{BI}_{it-1}+\beta_{3}{BD}_{it-1}+\beta_{4}{REM}_{it-1}+\beta_{5}{RPT}_{it-1}+\beta_{6}{LEV}_{it-1}+\beta_{7}{CA}_{it-1}+\beta_{8}{SIZE}_{it-1}+\varepsilon_{it}\;(\mathrm{Model}\;4b)$.

${EPS}_{it=}\alpha +\beta_{1}{BS}_{it-1}+\beta_{2}{BI}_{it-1}+\beta_{3}{BD}_{it-1}+\beta_{4}{REM}_{it-1}+\beta_{5}{RPT}_{it-1}+\beta_{6}{LEV}_{it-1}+\beta_{7}{CA}_{it-1}+\beta_{8}{SIZE}_{it-1}+\varepsilon_{it}\;(\mathrm{Model}\;4c)$.

${TQ}_{it=}\alpha +\beta_{1}{BS}_{it-1}+\beta_{2}{BI}_{it-1}+\beta_{3}{BD}_{it-1}+\beta_{4}{REM}_{it-1}+\beta_{5}{RPT}_{it-1}+\beta_{6}{LEV}_{it-1}+\beta_{7}{CA}_{it-1}+\beta_{8}{SIZE}_{it-1}+\varepsilon_{it}\;(\mathrm{Model}\;4d)$.

The estimation for lagged variables is consistent with the direct model outputs (Model 1) in Table 4 and with model (2) findings in Table 5.

#### 5.6.3. Heckman selection test

We have conducted several endogeneity tests in the earlier steps; however, there is a possibility of self-selection bias in the sample. This could be due to the BC and categories. Board independent members might not be a random choice of banks. Accordingly, we apply the Heckman two-stage test to address the issue of self-selection bias. We follow Westman [149], who studied corporate governance issues in European banks by including the previous year’s variables for the possible variables that may include the self-selection bias. Hence, in the initial stage of the Heckman test, we use the previous year’s data for BI. Then, we follow Kim et al. [150] by obtaining the fitted values of the dependent variables and using them as instrumental variables. In the next step, we follow Westman [149] by including two groups based on a categorical variable. We used BI as a categorical variable which denotes 1 if the bank has a proportion of more than 50% board independence and 0 otherwise. The following equation is followed for this purpose:

$${BICHDUMMY}_{it}=\gamma_{1}+\gamma_{2}{BS}_{it}+\gamma_{3}{BI}_{it}+\gamma_{4}{BICH}_{it}+\gamma_{5}{SIZE}_{it}+u_{it}$$ (12)

In all steps, the results in Table 9 show consistent and robust findings with the main models and 2SLS outcomes. Overall, the sample selection bias results align with the prior results’ estimation, suggesting that they are unlikely to be influenced by the potential self-selection bias.

**Table 9:** Two stage heckman test.

Variables	RONW	ROCE	EPS	PAT
C	-17.02***	-19.33***	-3.702***	35.089***
	12.95	6.972	9.324	36.922
BS	0.878**	0.549**	0.0803***	3.072**
	0.425	0.229	0. 257	1.211
BI	-2.927***	-4.094**	-8.183***	-44.918**
	9.576	5.149	0.529	27.320
BD	9.084***	9.051**	1.213***	52.737***
	9.021	4.852	0.506	25.735
REM	2.218***	2.158***	1.399**	0. 391***
	4.578	2.468	9.77–10	.00013
RPT	-2.335**	-1.455***	-2.646**	0.158***
	2.335	1.255	1.326	.0 666
LEV	-6.031***	-2.560***	0.429***	12.786
	1.665	.896	.092	4.749
CA	116.1	47.4	-1.885	6.148
	168.4	90.56	9.238	2.394
SIZE	-17.1***	-41.34***	-1.619	-27.011
	8.82	11.21	1.14	9.368
TYPE	13.011**	13.011**	-1.044*	13.011**
	5.325	5.325	6.625	5.325
BICH	-6.189***	-6.189***	2.036***	-6.189***
	2.397	2.397	.183	2.397
dummy	2.101***	2.101***	.367***	2.101***
	0.193	0.193	0.141	0.193
lambda	-5.972**	-2.921**	-2.008***	-17.496***
	2.348	1.266	.707	6.695
Dummy	-3.839***	-3.839***		-3.839***
	.343	.343		.343
Observations	366	366	366	366
Censored obs	251	251	251	251
Uncensored obs	115	115	115	115
Wald chi2(12)	51.85	41.55	34.08	67.25
Prob > chi2	0.000	0.000	0.000	0.000

Note

*** p<0.01

** p<0.05

* p<0.1. All variables are defined in Table 1.

### 5.7.Additional analysis

#### 5.7.1. The impact of RPTs and BC on FP on Public and Private Indian banks

We apply a bank-type dummy variable in which 1 denotes public banks, and 0 signifies private banks. We infer this step to estimate whether there is any difference between private and public banks. The following models are conducted:

$${FP}_{it}({ROA}_{it}/{PAT}_{it}/{EPS}_{it}/{TQ}_{it})=\alpha +\beta_{1}{BS}_{it}+\beta_{2}{BI}_{it}+\beta_{3}{BD}_{it}+\beta_{4}{REM}_{it}+\beta_{5}{RPT}_{it}+\beta_{6}{LEV}_{it}+\beta_{7}{CA}_{it}+\beta_{8}{SIZE}_{it}+\beta_{9}{TYPE}_{it}+\varepsilon_{it}$$ (Model 5)

The results in Table 10 provide an analysis of OLS regression for Model 5. The results show that bank type significantly impacts FP across the different measures. This indicates a significant positive difference at the level of 1% (þ < 0.01) between private and public banks.

**Table 10:** Impact of RPTs and BC on FP on Public and Private Indian banks.

Variables	RONW	ROCE	EPS	PAT
C	64.247**	24.191**	-12.585***	-9.200***
	24.295	11.763	25.613	22.400
BS	2.228***	1.290***	2.469***	15.332*
	0.420	0.198	0.415	6.391
BI	-11.836**	-5.354**	-8.460***	-18.520**
	5.681	2.691	3.382	27.020
BD	33.724***	18.358***	33.031**	33.720**
	8.463	3.983	9.296	13.360
REM	4.149**	3.279*	2.487**	4.579***
	1.228	0.143	0.256	1.243
RPT	2.034**	1.138***	1.830**	2.730***
	0.352	0.212	0.374	0.297
LEV	3.994***	0.905*	1.515	4.972**
	1.340	0.641	1.018	1.709
CA	-18.920*	-14.449***	6.082	8.781*
	51.450	24.185	2.663	3.810
SIZE	-19.806***	-9.313***	8.468**	8.494***
	3.222	1.558	4.014	3.062
TYPE	3.011***	3.482**	18.555***	9.157***
	4.639	2.247	3.401	4.832
R-squared	0.174	0.199	0.217	0.181
Adjusted R-squared	0.145	0.170	0.190	0.152
F-statistic	5.923	6.957	7.799	6.204
Prob(F-statistic)	0.000	0.000	0.000	0.000

Note

*** p<0.01

** p<0.05

* p<0.1. All variables are defined in Table 1.

${ROA}_{it=}\alpha +\beta_{1}{BS}_{it}+\beta_{2}{BI}_{it}+\beta_{3}{BD}_{it}+\beta_{4}{REM}_{it}+\beta_{5}{RPT}_{it}+\beta_{6}{LEV}_{it}+\beta_{7}{CA}_{it}+\beta_{8}{SIZE}_{it}+\beta_{9}{TYPE}_{it}+\varepsilon_{it}\;(\mathrm{Model}\;5a)$.

${PAT}_{it=}\alpha +\beta_{1}{BS}_{it}+\beta_{2}{BI}_{it}+\beta_{3}{BD}_{it}+\beta_{4}{REM}_{it}+\beta_{5}{RPT}_{it}+\beta_{6}{LEV}_{it}+\beta_{7}{CA}_{it}+\beta_{8}{SIZE}_{it}+\beta_{9}{TYPE}_{it}+\varepsilon_{it}\;(\mathrm{Model}\;5b)$.

${EPS}_{it=}\alpha +\beta_{1}{BS}_{it}+\beta_{2}{BI}_{it}+\beta_{3}{BD}_{it}+\beta_{4}{REM}_{it}+\beta_{5}{RPT}_{it}+\beta_{6}{LEV}_{it}+\beta_{7}{CA}_{it}+\beta_{8}{SIZE}_{it}+\beta_{9}{TYPE}_{it}+\varepsilon_{it}\;(\mathrm{Model}\;5c)$.

${TQ}_{it=}\alpha +\beta_{1}{BS}_{it}+\beta_{2}{BI}_{it}+\beta_{3}{BD}_{it}+\beta_{4}{REM}_{it}+\beta_{5}{RPT}_{it}+\beta_{6}{LEV}_{it}+\beta_{7}{CA}_{it}+\beta_{8}{SIZE}_{it}+\beta_{9}{TYPE}_{it}+\varepsilon_{it}\;(\mathrm{Model}\;5d)$.

The difference is for public banks compared to private banks, as bank type has a positive coefficient across all models. This indicates that the association between RPT and FP is greater in public banks than in private banks. Further, BI effectiveness in public banks weakens FP. Rao et al. [151] argued that foreign banks have higher ROA than public sector banks. Narwal and Pathneja [126] found that private-sector banks are more productive than public-sector banks due to better technology utilization. However, the profitability of the two bank groups is not significantly different.

#### 5.7.2. Alternative measures of FP

We apply an alternative measure for FP using ROCE. We use this alternative measure of financial performance to investigate the robustness of our results. This is to ensure the benchmark results were not affected by other indicators to measure corporate financial performance [152]. Several studies have used different measures for banks’ financial performance. For example, Naeem et al. [153] and Rani and Zergaw [154] used (ROA). Similarly, Garcia and Guerreiro [155] and Yahya et al. [156] measured the financial performance of banks using ROE. In the same quest, Al-Homaidi et al. [40] used NIM. However, the present study uses the ROCE of banks as an alternative measure of FP. The model is as follows:

$${ROCE}_{it=}\alpha +\beta_{1}{BS}_{it}+\beta_{2}{BI}_{it}+\beta_{3}{BD}_{it}+\beta_{4}{REM}_{it}+\beta_{5}{RPT}_{it}+\beta_{6}{LEV}_{it}+\beta_{7}{CA}_{it}+\beta_{8}{SIZE}_{it}+\varepsilon_{it}$$ (Model 6)

The estimation is conducted in three steps. First, we apply a random effect model for the explanatory and control variables. Second, we add a dummy variable of bank type to assess if there are differences between the two types of banks.


$${ROCE}_{it=}\alpha +\beta_{1}{BS}_{it}+\beta_{2}{BI}_{it}+\beta_{3}{BD}_{it}+\beta_{4}{REM}_{it}+\beta_{5}{RPT}_{it}+\beta_{6}{LEV}_{it}+\beta_{7}{CA}_{it}+\beta_{8}{SIZE}_{it}+\beta_{9}{TYPE}_{it}+\varepsilon_{it}$$ (Model 6A)

Finally, we infer the results using the moderating effect of BICH. To estimate this step, we apply the following model:

$${ROCE}_{it=}\alpha +\beta_{1}{BS}_{it}+\beta_{2}{\left({BS}_{}\;x\;{BICH}_{}\right)}_{it}+\beta_{3}{BICH}_{it}+\beta_{4}{BD}_{it}+\beta_{5}{\left({BD}_{}\;x\;{BICH}_{}\right)}_{it}+\beta_{6}{BREM}_{it}+\beta_{7}{\left({REM}_{}\;x\;{BICH}_{}\right)}_{it}+\beta_{8}{RPT}_{it}+\beta_{9}{\left({RPT}_{}\;x\;{BICH}_{}\right)}_{it}+\beta_{10}{LEV}_{it}+\beta_{11}{\left({LEV}_{}\;x\;{BICH}_{}\right)}_{it}+\beta_{12}{CA}_{it}+\beta_{13}{\left({CA}_{}\;x\;{BICH}_{}\right)}_{it}+\beta_{14}{SIZE}_{it}+\beta_{15}{\left({SIZE}_{}\;x\;{BICH}_{}\right)}_{it}+\varepsilon_{it}$$ (Model 6B)

The findings presented in Table 11 estimate the direct, dummy, and moderating models for the alternative measure of financial performance. Interestingly, the results of board characteristics, related party transactions, and banks’ specifics demonstrate similar findings to the earlier models conducted in Tables 4 and 5, except for some variations in the significance levels across some variables; however, these variations do not affect the findings. The findings yielded from these estimations are robust and consistent with the findings of the main and moderating models conducted previously. This implies that our analysis of the direct models (Table 4) using a different measure for FP (*ROA*_*it*_/*PAT*_*it*_/*EPS*_*it*_/*TQ*_*it*_) and the moderating effect (Table 5) is, therefore, robust and not sensitive to a specific measure of financial performance.

**Table 11:** An alternative measure of financial performance.

Variables	Direct	TYPE	BICH
*C*	8.825***	9.120***	29.620***
	2.094	2.697	4.025
*BS*	0.088***	0.088***	0.381***
	0.015	0.016	0.080
*BSx BICH*			-0.440**
			0.119
*BICH*	-0.674**	-0.638**	-1.709***
	0.258	0.242	1.028
*BD*	1.390***	1.391***	5.801**
	0.339	0.341	2.058
*BDx BICH*			-6.731**
			2.999
*REM*	5.217**	2.934***	7.069**
	2.824	3.312	1.405
*REMx BICH*			6.406***
			4.272
*RPT*	-3.395***	-0.391**	1.096***
	13.892	6.791	20.400
*RPTx BICH*			2.221***
			0.497
*LEV*	0.322**	0.323**	1.126***
	0.160	0.161	0.277
*LEVx BICH*			-1.221***
			0.380
*CA*	2.047	1.951	-2.894***
	4.341	4.320	6.560
*CAx BICH*			-3.012
			15.397
*SIZE*	-1.775***	-1.809***	-6.371***
	0.415	0.451	0.628
*SIZEx BICH*			6.522***
			0.837
*TYPE*		-0.075***	
		0.371	
R-squared	0.291	0.291	0.250
Adjusted R-squared	0.268	0.266	0.214
F-statistic	12.537	11.513	12.611
Prob(F-statistic)	0.000	0.000	0.000

Note

*** p<0.01

** p<0.05

* p<0.1. All variables are defined in Table 1.

#### 5.7.3. The impact of RPTs and BC on FP of Indian banks pre- and post-Indian accounting standard

To assess the impact of Ind. ASs, we use a dummy variable of 1 for the period after the implementation of Ind. ASs in 2017 onwards and 0 otherwise. Accordingly, we apply the following model:

$${FP}_{it}({ROA}_{it}/{PAT}_{it}/{EPS}_{it}/{TQ}_{it})=\alpha +\beta_{1}{BS}_{it}+\beta_{2}{BI}_{it}+\beta_{3}{BD}_{it}+\beta_{4}{REM}_{it}+\beta_{5}{RPT}_{it}+\beta_{6}{LEV}_{it}+\beta_{7}{CA}_{it}+\beta_{8}{SIZE}_{it}+\beta_{9}{Ind.Ss}_{it}+\beta_{10}{TYPE}_{it}\varepsilon_{it}$$ (Model 7)

The results in Table 12 provide an analysis of OLS regression for Model 7. The results show that Ind. ASs show a significant negative effect at 1% (þ < 0.01) across the models. This indicates that there is a significant change between pre and post-Ind. ASs. Importantly, the results show that BC exhibit higher significance than the earlier results. However, the effect of BC and RPT are consistent with the results in Model 1. Further, the results show that bank type exhibits a significant impact on FP. This indicates a significant positive difference between private and public banks. Public banks exhibit better FP, which is indicated by a positive coefficient of bank type. In the same context, the results show FP is better in pre-Ind. ASs. as compared to post-Ind. ASs which is indicated by a negative coefficient of Ind. ASs.

**Table 12:** Alternative measure of financial performance.

Variables	Model (7a)	Model (7b)	Model (7c)	Model (7d)
C	24.507	2.403	-130.276	-18.000***
	25.665	13.792	84.773	44.880
BS	1.276***	0.824***	1.171**	11.095***
	0.369	0.207	1.012	4.210
BI	-10.225***	-4.703***	-12.332**	-6.620**
	3.472	1.449	8.982	2.332
BD	21.815***	12.730***	18.863*	15.300*
	5.523	3.110	12.331	8.813
REM	26.228***	14.126***	30.744***	22.228**
	5.824	3.095	9.197	9.824
RPT	1.320***	1.013**	9.391**	4.267***
	1.505	0.588	2.326	1.218
LEV	1.392***	-0.363**	-4.384****	7.186**
	1.822	0.720	2.173	2.362
CA	-1.852**	-33.628**	8.155*	4.600*
	4.573	28.720	3.216	2.900
SIZE	-9.396***	-3.934**	15.853*	8.000***
	4.058	1.594	3.252	2.103
Ind. ASs	-21.398***	-10.112***	-35.223***	-9.640***
	5.743	2.514	10.696	3.866
TYPE	3.606**	4.048*	14.530**	7.000*
	6.046	2.957	7.301	2.512
R-squared	0.292	0.164	0.200	0.258
Adjusted R-squared	0.268	0.152	0.189	0.233
F-statistic	16.743	17.657	6.498	14.471
Prob(F-statistic)	0.000	0.000	0.000	0.000

Note

*** p<0.01

** p<0.05

* p<0.1. All variables are defined in Table 1.

${ROA}_{it=}\alpha +\beta_{1}{BS}_{it}+\beta_{2}{BI}_{it}+\beta_{3}{BD}_{it}+\beta_{4}{REM}_{it}+\beta_{5}{RPT}_{it}+\beta_{6}{LEV}_{it}+\beta_{7}{CA}_{it}+\beta_{8}{SIZE}_{it}+\beta_{9}{Ind.Ss}_{it}+\beta_{10}{TYPE}_{it}{+\varepsilon }_{it}\;(\mathrm{Model}\;7a)$

${PAT}_{it=}\alpha +\beta_{1}{BS}_{it}+\beta_{2}{BI}_{it}+\beta_{3}{BD}_{it}+\beta_{4}{REM}_{it}+\beta_{5}{RPT}_{it}+\beta_{6}{LEV}_{it}+\beta_{7}{CA}_{it}+\beta_{8}{SIZE}_{it}+\beta_{9}{Ind.Ss}_{it}+\beta_{10}{TYPE}_{it}{+\varepsilon }_{it}\;(\mathrm{Model}\;7b)$.

${EPS}_{it=}\alpha +\beta_{1}{BS}_{it}+\beta_{2}{BI}_{it}+\beta_{3}{BD}_{it}+\beta_{4}{REM}_{it}+\beta_{5}{RPT}_{it}+\beta_{6}{LEV}_{it}+\beta_{7}{CA}_{it}+\beta_{8}{SIZE}_{it}+\beta_{9}{Ind.Ss}_{it}+\beta_{10}{TYPE}_{it}{+\varepsilon }_{it}\;(\mathrm{Model}\;7c)$.

${TQ}_{it=}\alpha +\beta_{1}{BS}_{it}+\beta_{2}{BI}_{it}+\beta_{3}{BD}_{it}+\beta_{4}{REM}_{it}+\beta_{5}{RPT}_{it}+\beta_{6}{LEV}_{it}+\beta_{7}{CA}_{it}+\beta_{8}{SIZE}_{it}+\beta_{9}{Ind.Ss}_{it}+\beta_{10}{TYPE}_{it}{+\varepsilon }_{it}\;(\mathrm{Model}\;7d)$.

## 6. Conclusion

The current study investigates the moderating effect of board independence change on the relationship between board characteristics, related party transactions, and financial performance of Indian listed banks. While board size, independence, diligence, and remuneration were taken to represent board characteristics, all key personnel and all subsidiaries were considered as measures for related party transactions. On the other hand, banks’ financial performance was measured using accounting-based measures (return on assets and profit after tax) and market-based measures (earning per share and Tobin Q). In this comprehensive research model, we examined the effect of RPTs and BC on the FP in a sample comprising 38 Indian public and private banks from 2010 to 2019 using several statistical analysis tools. In the first analysis stage, we estimated the impact of board characteristics and related party transactions on banks’ financial performance. In the second step, we estimated the moderation role of board independence change on the relationship between related party transactions and board characteristics on banks’ financial performance. We found that board independence change has a significant negative influence on financial performance. Further, the results indicate that board independence change moderates positively and significantly the relationship between related party transactions and financial performance. The results also show that board independence change has a moderating effect that weakens significantly and negatively affects board size and effectiveness, negatively influencing banks’ profitability. Consequently, in the third stage of analysis, several robustness checks have been conducted using multiple tools of analysis that include two-stage least square regression, generalized method of moments, lag of independent variables, and Heckman selection test. In this step, the results showed consistent and robust findings with the main outcomes. Finally, in the last stage of our analysis, we provided some additional analysis to test the sensitivity of the results. In this stage, we estimated the impact of related party transactions and board characteristics on banks financial performance of private and public banks.

Then, we also use an alternative measure of financial performance to test the sensitivity of the results provided in the preceding steps. Finally, we estimate the impact of related party transactions and board characteristics on financial performance, taking into consideration the effect of Ind. ASs in which we conducted the pre-post analysis. In this step, the results show that bank type significantly impacts FP across the different measures. This indicates a significant positive difference at the level of 1% (þ < 0.01) between private and public banks. The results show that bank type exhibit a significant impact on FP. And public banks exhibit better FP, especially pre-Ind. ASs.

As a result, the current study adds to the existing literature and fills a gap in research studies on the banking sector in India. In the Indian context, there is no evidence on this topic for the banking sector. Few studies have investigated the relationship between RPTs and bank financial performance in some developed countries; however, the unique institutional, legal and financial settings in these countries differ from those in emerging countries, particularly India. As a result, this could have both practical and theoretical implications for other emerging economies. The current study also makes a unique contribution in that it investigates how all key personnel and subsidiaries, acting as proxies for RPTs, influence bank financial performance in India by influencing board size, board composition, and board remuneration. Finally, the study examines the moderating effect of board independence change on the relationship between related party transactions and board characteristics on bank financial performance, which has previously been overlooked in research.

As a result, the current study provides valuable insights into several issues concerning current RPT and BC practices in Indian banks. Bankers, regulators, and policymakers are provided with valuable insights for improving the performance of Indian banks and controlling the negative aspects of RPTs. Unlike other studies, this study uses board independence change as a moderator between board characteristics, related party transactions, and financial performance measures. Limited research highlights this issue where Indian banks have encountered several challenges in the last few years. Non-performing assets, competition from the non-banking sector and foreign banks, bureaucracy, and political influence are some significant challenges to Indian banks. Further, various types of fraud were witnessed in India’s banking industry. “Punjab and Maharashtra Co-operative banks” witnessed fraudulent lending practices in November 2019. YES Bank, a private bank, was put under the control of the Reserve Bank of India due to high bad loans to avoid collapse. Accordingly, the present study is motivated by this background to bridge the gaps in the strand literature and bring empirical evidence on Indian banks. As a result, this study provides useful insights and has implications for policymakers, bankers, financial analysts, and academics.

This study has some limitations that may guide possible future research. First, the study did not include ownership and audit committee variables due to the non-availability of data. Therefore, it is suggested that future studies consider changes in audit committee attributes and include different categories of ownership structure. Second, this study presents empirical evidence from a developing country; hence other studies may wish to investigate and compare the evidence from developed and developing countries. Another possible stream of research is comparing different sectors and industries on this issue.
