# Correction: The moderation role of board independence change in the relationship between board characteristics, related party transactions, and financial performance

**DOI:** 10.1371/journal.pone.0324642

**Published:** 2025-05-20

**Authors:** Faozi A. Almaqtari, Najib H. S. Farhan, Hamood Mohammed Al-Hattami, Tamer Elsheikh

There is an error in affiliation 2 for author Najib H. S. Farhan. The correct affiliation 2 is: Faculty of Business Studies, Arab Open University, Kingdom of Saudi Arabia.
